# Erratum to: An overview of the National COVID-19 Chest Imaging Database: data quality and cohort analysis

**DOI:** 10.1093/gigascience/giab083

**Published:** 2021-12-01

**Authors:** Dominic Cushnan, Oscar Bennett, Rosalind Berka, Ottavia Bertolli, Ashwin Chopra, Samie Dorgham, Alberto Favaro, Tara Ganepola, Mark Halling-Brown, Gergely Imreh, Joseph Jacob, Emily Jefferson, François Lemarchand, Daniel Schofield, Jeremy C Wyatt, N C C I D Collaborative

**Affiliations:** AI Lab, NHSX, Skipton House, 80 London Road, London SE1 6LH, UK; Faculty, 54 Welbeck Street, London W1G 9XS, UK; Faculty, 54 Welbeck Street, London W1G 9XS, UK; Faculty, 54 Welbeck Street, London W1G 9XS, UK; Faculty, 54 Welbeck Street, London W1G 9XS, UK; Faculty, 54 Welbeck Street, London W1G 9XS, UK; Faculty, 54 Welbeck Street, London W1G 9XS, UK; Faculty, 54 Welbeck Street, London W1G 9XS, UK; Scientific Computing, Royal Surrey NHS Foundation Trust, Egerton Road, Guildford GU2 7XX, UK; Faculty, 54 Welbeck Street, London W1G 9XS, UK; UCL Respiratory, 1st Floor, Rayne Institute, University College London, London WC1E 6JF, UK; Health Data Research UK, Gibbs Building, 215 Euston Road, London NW1 2BE, UK; Health Informatics Centre (HIC), School of Medicine, University of Dundee, DD1 4HN, Dundee, UK; AI Lab, NHSX, Skipton House, 80 London Road, London SE1 6LH, UK; AI Lab, NHSX, Skipton House, 80 London Road, London SE1 6LH, UK; Emeritus Professor of Digital Healthcare, University of Southampton, Southampton SO17 1BJ, UK; NHSX, Skipton House, 80 London Road, London SE1 6LH, UK; NCCID Collaborative

## Correction

In the final publication of “An overview of the National COVID-19 Chest Imaging Database: data quality and cohort analysis” by Dominic Cushnan et al., figure 9 was erroneously duplicated in place of figure 8. Figure 8 (“comparison of sex split within (A) the NCCID COVID-19 patients, the general UK population (as reported in the 2011 census), and COVID-19 hospital admissions (reported by ISARIC); (B) NCCID recorded deaths and NHS England COVID-19 hospital mortality data”) now appears correctly in the article.

The Publisher regrets this error.

