# Supplementary material for: An overview of the National COVID-19 Chest Imaging Database: data quality and cohort analysis
Source: Gigascience. 2021 Nov 25;10(11):giab076. doi: 10.1093/gigascience/giab076 (PMC8633457; doi:10.1093/gigascience/giab076)
Supplement: giab076_GIGA-D-21-00096_Original_Submission [file giab076_giga-d-21-00096_original_submission.pdf]

GigaScience

An overview of the National COVID-19 Chest Imaging Database: data quality and cohort analysis

--Manuscript Draft--

**Manuscript Number:**	GIGA-D-21-00096
**Full Title:**	An overview of the National COVID-19 Chest Imaging Database: data quality and cohort analysis
**Article Type:**	Data Note
**Funding Information:**	NHSX — Not applicable
**Abstract:**	Background: The National COVID-19 Chest Imaging Database (NCCID) is a centralised database containing chest X-rays, Computed Tomography (CT) scans and cardiac Magnetic Resonance Images (MRI) from patients across the UK. The objective of the initiative is to support a better understanding of the coronavirus SARS-CoV-2 disease (COVID-19) and the development of machine learning technologies that will improve care for patients hospitalised with a severe COVID-19 infection. The NCCID is now accumulating data from 20 NHS sites across England and Wales, with a total contribution of approximately 25,000 imaging studies in the training set (at time of writing) and is actively being used as a research tool by several organisations. Findings: This paper introduces the training dataset, including a snapshot analysis covering: the completeness of clinical data, and availability of image data for the various use-cases (diagnosis, prognosis, longitudinal risk). Findings suggests the NCCID is well suited for developing clinical models, but developers<br><br>should take care to mitigate the common model confounders, e.g., equipment type, that are highlighted. In addition, a cohort analysis was performed to measure the representativeness of the NCCID to the wider COVID-19 affected population. Three major aspects were included: geographic, demographic and temporal coverage, revealing good alignment in some categories, e.g., sex, whilst also identifying areas for improvements to data collection methods, particularly with respect to geographic coverage.<br><br>Conclusion: The NCCID is a growing resource that provides researchers with a large, high-quality database that can be leveraged to support the response to the COVID-19 pandemic.
**Corresponding Author:**	Dominic Cushnan<br>NHS AI Lab, NHSX<br>London, UNITED KINGDOM
**Corresponding Author Secondary Information:**	
**Corresponding Author's Institution:**	NHS AI Lab, NHSX
**Corresponding Author's Secondary Institution:**	
**First Author:**	Dominic Cushnan
**First Author Secondary Information:**	
**Order of Authors:**	Dominic Cushnan<br>Oscar Bennett<br>Rosalind Berka<br>Ottavia Bertolli<br>Ashwin Chopra<br>Samie Dorgham

Powered by Editorial Manager® and ProduXion Manager® from Aries Systems Corporation

	Alberto Favaro
	Tara Ganepola
	Mark Halling-Brown
	Gergely Imreh
	Joseph Jacob
	Emily Jefferson
	Francois Lemarchand
	Daniel Schofield
	Jeremy C. C. Wyatt
	NCCID Collaborative
**Order of Authors Secondary Information:**	
**Additional Information:**	
**Question**	**Response**
Are you submitting this manuscript to a special series or article collection?	No
**Experimental design and statistics**<br><br>Full details of the experimental design and statistical methods used should be given in the Methods section, as detailed in our Minimum Standards Reporting Checklist. Information essential to interpreting the data presented should be made available in the figure legends.<br><br>Have you included all the information requested in your manuscript?	Yes
**Resources**<br><br>A description of all resources used, including antibodies, cell lines, animals and software tools, with enough information to allow them to be uniquely identified, should be included in the Methods section. Authors are strongly encouraged to cite Research Resource Identifiers (RRIDs) for antibodies, model organisms and tools, where possible.<br><br>Have you included the information requested as detailed in our Minimum Standards Reporting Checklist?	Yes

Powered by Editorial Manager® and ProduXion Manager® from Aries Systems Corporation

**Availability of data and materials**<br><br>All datasets and code on which the conclusions of the paper rely must be either included in your submission or deposited in publicly available repositories (where available and ethically appropriate), referencing such data using a unique identifier in the references and in the “Availability of Data and Materials” section of your manuscript.<br><br>Have you have met the above requirement as detailed in our Minimum Standards Reporting Checklist?	No
If not, please give reasons for any omissions below.<br><br>as follow-up to "**Availability of data and materials**<br><br>All datasets and code on which the conclusions of the paper rely must be either included in your submission or deposited in publicly available repositories (where available and ethically appropriate), referencing such data using a unique identifier in the references and in the “Availability of Data and Materials” section of your manuscript.<br><br>Have you have met the above requirement as detailed in our Minimum Standards Reporting Checklist?<br><br>"	Access to the dataset can be sought via an application to the National COVID-19 Chest Imaging Database (NCCID) Data Access Committee as described on the NCCID website linked.<br><br>https://nhsx.github.io/covid-chest-imaging-database/

Powered by Editorial Manager® and ProduXion Manager® from Aries Systems Corporation

Manuscript

Click here to access/download;Manuscript;NCCID_GS_version1_0_submiss

GigaScience, 2021, 1–19

doi: xx.xxxx/xxxx
Manuscript in Preparation
Data Note

DATA NOTE

An overview of the National COVID-19 Chest Imaging Database: data quality and cohort analysis

Dominic Cushnan[1], Oscar Bennett[2], Rosalind Berka[2], Ottavia Bertolli[2], Ashwin Chopra[2], Samie Dorgham[2], Alberto Favaro[2], Tara Ganepola[2], Mark Halling-Brown[3], Gergely Imreh[2], Joseph Jacob[4,5], Emily Jefferson[6,7], François Lemarchand[1], Daniel Schofield[1], Jeremy C Wyatt[8,9] and NCCID Collaborative[10]

[1]AI Lab, NHSX, Skipton House, 80 London Road, London SE1 6LH and [2]Faculty, 54 Welbeck Street, London W1G 9XS and [3]Scientific Computing, Royal Surrey NHS Foundation Trust, Egerton Road, Guildford, GU2 7XX and [4]UCL Respiratory, 1st Floor, Rayne Institute, University College London, London, WC1E 6JF and [5]UCL Respiratory, 1st Floor, Rayne Institute, University College London, London, WC1E 6JF and [6]Health Data Research UK, Gibbs Building, 215 Euston Road, London, NW1 2BE and [7]Health Informatics Centre (HIC), School of Medicine, University of Dundee, DD1 4HN and [8]Emeritus Professor of Digital Healthcare, University of Southampton, Southampton SO17 1BJ and [9]Advisor, NHSX, Skipton House, 80 London Road, London SE1 6LH and [10]NCCID Collaborative

*Corresponding author: francois.lemarchand@nhsx.nhs.uk

Abstract

Background: The National COVID-19 Chest Imaging Database (NCCID) is a centralised database containing chest X-rays, Computed Tomography (CT) scans and cardiac Magnetic Resonance Images (MRI) from patients across the UK. The objective of the initiative is to support a better understanding of the coronavirus SARS-CoV-2 disease (COVID-19) and the development of machine learning technologies that will improve care for patients hospitalised with a severe COVID-19 infection. The NCCID is now accumulating data from 20 NHS sites across England and Wales, with a total contribution of approximately 25,000 imaging studies in the training set (at time of writing) and is actively being used as a research tool by several organisations. **Findings:** This paper introduces the training dataset, including a snapshot analysis covering: the completeness of clinical data, and availability of image data for the various use-cases (diagnosis, prognosis, longitudinal risk). Findings suggests the NCCID is well suited for developing clinical models, but developers should take care to mitigate the common model confounders, e.g., equipment type, that are highlighted. In addition, a cohort analysis was performed to measure the representativeness of the NCCID to the wider COVID-19 affected population. Three major aspects were included: geographic, demographic and temporal coverage, revealing good alignment in some categories, e.g., sex, whilst also identifying areas for improvements to data collection methods, particularly with respect to geographic coverage. **Conclusion:** The NCCID is a growing resource that provides researchers with a large, high-quality database that can be leveraged to support the response to the COVID-19 pandemic.

Key words: SARS-CoV2; COVID-19; thoracic imaging; medical imaging; machine learning;

Compiled on: April 2, 2021.
Draft manuscript prepared by the author.

Background

Radiology has played a significant role during the pandemic, informing our understanding of the COVID-19 disease [1, 2, 3, 4] and guiding decision making along care pathways. Clinicians have identified characteristic features of COVID-19 acute respiratory distress from thoracic imaging studies; such features can be used to differentiate COVID-19 patients from those suffering other respiratory conditions [3, 5, 6]. However, these differences in disease manifestation are often subtle [7] and may be more quantitatively delineated using computational methods.

One corollary of the widespread adoption of radiology during the pandemic is the accumulation of large volumes of clinical imaging data spread across hospital sites throughout the UK. The National COVID-19 Chest Imaging Database (NCCID) was established to collate this mass of X-ray, CT and MRI scans into an accessible imaging database. The end goal of the NCCID is to facilitate researchers and technology developers in the creation of fair, effective and generalisable machine learning (ML) technologies that can support diagnosis, prognosis and risk stratification of the COVID-affected population, ultimately aiding clinicians to improve patient outcomes.

The initiative was formed as part of the NHS AI lab's mission of enabling the safe adoption of AI technologies in the NHS [8] and was successfully set up through partnerships with the Royal Surrey NHS Foundation Trust (RSNFT), the British Society of Thoracic Imaging (BSTI) and Faculty, an AI technology company. This combination of data processing and clinical expertise has been leveraged to create a data warehouse comprising pseudonymised thoracic imaging and relevant clinical data points for thousands of patients across the UK. Further information on the NCCID's remit and rationale are described in an article in the European Respiratory Journal [9].

A portion of the data is transferred to the training set, which contained 24,465 imaging studies from 7,685 patients at time of writing (latest figures can be found on the NCCID information page). The remaining portion of data is allocated to the validation set, which is protected as a hold-out set for NHSX to conduct future performance assessments of COVID-19 chest-imaging AI technologies, ensuring that they are safe and effective before testing in a real-world clinical setting. Results presented in this paper are solely focused on the training data, in order to maintain the integrity of the validation data as a hold-out benchmarking tool.

This article aims to describe key characteristics of the data and indicate its usefulness for developing algorithms that can support COVID-19 diagnosis and prognosis from chest images. The work was conducted on pseudonymised data within the existing NHSE AWS cloud infrastructure for the NCCID. To preserve the privacy of individuals, suppression of small numbers has been implemented throughout the paper. Suppressed data is indicated within plots and tables by the presence of an asterisk (*) for categories containing less than 7 individuals.

As the data is submitted in two parts - the images themselves, and the clinical data separately - the analysis has naturally been structured in this manner with an additional investigation of how the geographic, demographic and temporal coverage of the dataset compares with publicly available datasets for the wider COVID-affected population. The implications of these findings for developing algorithms related to COVID-19 is provided in the Discussion, alongside a list of future aims that have been identified to improve the dataset.

Methods

Database Setup

Figure 1 provides an overview of the data collection pipeline for the NCCID warehouse, which can be broadly broken down into the following stages:

i. NCCID participating collection sites (hospitals) are requested to contribute imaging data for patients that have undergone a real-time Reverse Transcription Polymerase Chain Reaction (RT-PCR) test for COVID-19. In addition to the images, two spreadsheets with different fields for the positive and negative cases are populated to capture accompanying clinical data (see clinical data and supplementary resources for more information).

ii. The Scientific Computing Team at RSNFT have established a dedicated node on Sectra's Image Exchange Portal (IEP) for receiving the images. IEP is a widely used network for sharing images between hospitals. The images are received by a SMART (Secure Medical-Image Anonymiser Receiver for Trials) box in Random Access Memory (RAM) and de-identified before writing to disk, ensuring that no patient identifiable information leaves the sites. The clinical data spreadsheet is also de-identified by means of a common pseudonym, generated via a one-way hashing algorithm combined with a complex salt and uploaded to a

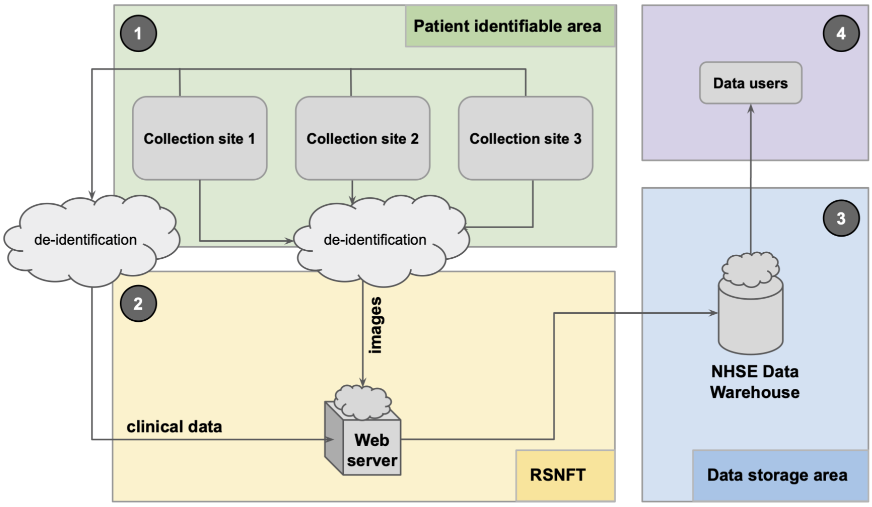

Figure 1. Diagram of the data collection pipeline for the NCCID warehouse.

web portal. Upon receiving images and clinical information, RSNFT links the two sources using the pseudonym. Patient's unique digital identifiers (NHS number or equivalent for devolved nations) are also encrypted using an Advanced Encryption Standard (AES) algorithm and a complex salt to allow linkage with other national-level datasets.

iii. The data is transferred to a central NCCID data warehouse hosted inside NHS England's (NHSE) Amazon Web Services (AWS) infrastructure, designed and implemented by Faculty and NHXS. The warehouse is backed by a single Simple Storage Service (S3) bucket within a separate sub-account under NHSE's AWS organisation. All data within the S3 bucket is encrypted at rest using AES-256 encryption. Data is regularly split into training and validation sets based on a randomisation of patients: once a patient has entered the training or validation set, any new images for that patient are automatically added to the same set. The codebase for warehouse infrastructure is open-source (see Code Availability).

iv. Data users that have been approved through the DAR process can access the training set. Image files are available in DICOM format, and clinical data is stored as JSON files. AWS credentials for the S3 bucket are provided to an organisation via an encrypted communication. Further support, including guidelines and code for access the data are provided through the information site

Inclusion Criteria

The inclusion criteria for individuals within the NCCID database are as follows:

- The person has undergone a COVID-19 swab test (RT-PCR). The outcome of the test may have been positive or negative. Some individuals may have undergone multiple swab tests;
- The person has undergone chest imaging in the three weeks before or after the swab.

The positive cohort consists of the individuals that returned one or more positive swab tests. All imaging data associated with a positive patient's COVID-19 hospital episode have been requested. To provide insight on longitudinal risk factors, historical images up to January 2017 are also requested.

The negative cohort consists of individuals for whom all acquired swab tests return negative. This may differ from some clinical databases where the control cohort represents healthy individuals but was deemed the correct method for curating a dataset that could train the most useful diagnostic models that differentiate COVID-19 characteristic features from other respiratory conditions. Thoracic images acquired within the six-week window surrounding the test are requested.

Although the status of a patient's RT-PCR swab test serves as a proxy for ground truth, users should be aware of the limitations of these labels. In particular, this method of testing has a relatively low sensitivity score, where estimates range from 0.71-0.98 [10], this causes the false omission rate to be quite high. In addition, the probability of having a COVID-19 infection is higher in those attending hospital with respiratory symptoms, than for the general public. Given these factors, data users should expect the negative cohort to contain a non-negligible portion of mislabelled positive patients. Additional clinical assessment of the images may be required to improve the accuracy of labels.

Imaging Data

The NCCID is a continually growing asset, as such, all subsequent figures and analyses reported in this paper refer to the training data as of 29 October 2020 (unless otherwise stated). On this date, the NCCID training dataset contained data for 7,500 patients; Table 1 details how this cohort is split by control/disease and data availability. There were 1,307 patients with clinical data only due to the fact that the accompanying images had not yet been uploaded by the PACS teams.

Table 2 details the image modality breakdown for the patients that have had their imaging data uploaded to the training dataset. The majority of the image studies (see glossary in Appendix A for definition) in the NCCID are X-rays, followed by CTs. Only a small number of MRIs, 17, have been submitted, therefore MRI data is excluded from further analysis. A single patient may have multiple studies within the NCCID, for instance, if multiple diagnostic scans were taken during their treatment pathway or historic scans were provided (see image characteristics section for more details).

Clinical Data

The NCCID sites have been asked to provide additional clinical information alongside imaging data for any patients that have tested positively for COVID-19 via the RT-PCR swab test. The intended purpose of this additional information is to provide researchers with insight into potential causal risk factors, such as comorbidities, as well as potential variables that indicate severity of disease. The clinical data can be broken down into five broad categories:

i. *Demographic information* – age, sex, ethnicity. This data is discussed in detail in the demographics section.

ii. *Important dates* – such as swab dates, image dates and date of admission.

iii. *Patient medical history*, specifying any pre-existing conditions, and the current use of some drugs such as blood pressure medications.

iv. *Admission metrics*, detailing the condition of the patient on admission to hospital i.e., blood pressure, lymphocyte count, partial pressure of O2 etc.

v. *COVID information*, pertaining to how the patient was treated (intubation, admitted to ITU), the results of their RT-PCR-tests, the severity associated with their chest X-ray [11], and their ultimate COVID and mortality status.

For patients in the control cohort, only a subset of this information was requested: patient pseudonym, submitting centre, date of RT-PCR, and result of RT-PCR. This decision was made to reduce the burden on busy ward staff during the pandemic. Schemas for both spreadsheets are available through the supplementary resources section.

Initial investigation of the clinical data revealed several data quality issues, as can be expected during a pandemic when resources and time are understandably limited. Issues included: non-numeric values, such as blank spaces reported for numeric fields; inconsistency of date/time formats with some entries in US (month-day-year) versus UK (day-month-year) format; mismatch in format for reporting categorical data (e.g., M, F for Male, Female versus 0, 1); different sites using different unit scales to report clinical metrics, e.g., mg/L versus ng/L. To address many of these issues a data cleaning pipeline was created and made publicly available to data users, alongside additional details on the data quality issues, and guidance on the expected format of the clinical data fields (see supplementary resources section).

Missing values in the demographic data were backfilled using a segmentation dataset provided by NHS England and Improvement (NHSEI) for ethnicity data (internal resource, citation pending), and DICOM header information for sex and

Table 1. Breakdown of patient cohorts

PCR-RT swab status:	Patients with images and clinical data:	Patients with clinical data only	Totals:
Positive patients	2,881	287	**3,168**
Negative patients	3,312	1,020	**4,332**
**Totals:**	**6,193**	**1,307**	**7,500**

Table 2. Modality breakdown of image studies by patient cohort

PCR-RT swab status	No. of X-ray studies	No. of CT studies	Totals
Positive patients	11,725	1,565	**13,294**
Negative patients	5,532	1,112	**6,651**
**Totals:**	**17,257**	**2,677**	**19,945**

age. Making these sensitive attributes available to users is vital for measuring and facilitating equality of care, particularly through bias mitigation of ML models. As such, the additional source of ethnicity data has also been made available to data users.

The results that are reported in this paper are based on the cleaned data for which known errors, such as non-numerical entries have been removed. Text input has been parsed to extract embedded numeric values, and categorical values have been mapped to standard schemas. Issues arising from ambiguous dates (i.e., 03/04 vs 04/03) and mixed measurement units have not been fully rectified by the cleaning pipeline and may persist.

Data Validation

The following analyses are provided to aid data users in understanding the suitability of the NCCID training dataset for developing diagnostic and prognostic algorithms based on COVID-19 chest imaging:

i. *Clinical data completeness*: assess the completeness and quality of the clinical data, particularly in relation to pertinent information (e.g., comorbidities, disease severity, outcomes) that can provide additional training variables or labels for ML models.
ii. *Imaging characteristics*: considers the availability of historical data for longitudinal studies, the implications of the timing of image acquisition along care pathways, and potential model confounders such as the scanner type.
iii. *Cohort analysis*: to inform NCCID users of any potential biases in the training dataset that could impede their ability to develop fair, effective, and generalisable AI models. To achieve this, we compared the geographic, demographic, and temporal distributions of patients in the NCCID with publicly available datasets, measuring how far the data is representative of the wider population that has been affected by COVID-19.

The subsequent sections follow the structure of the above three categories, each containing a description of the methodology (if applicable) alongside the key results. The implications of these findings for building ML models are elaborated in the discussion section.

Clinical data completeness

To understand the utility and limitations of the clinical data with respect to developing diagnostic or prognostic AI models, we assessed the completeness of each field in the four categories: important dates, patient medical history, admission metrics, and COVID information. Completeness was quantified in terms of the percentage of null and not-null values submitted for each field across all COVID-positive patients.

Figure 2 A-D show the completeness of the clinical data after applying the cleaning pipeline (see the clinical data methodology section). For each field of the clinical data, the percentage of entries with non-null values are shown in orange against the percentage of null values in blue. The data exhibits varying degrees of completeness with several well-reported fields present in over 80% of patients, but the majority of fields are between 0%-50% complete. The subsequent subsections investigate each plot more closely.

Dates

The date of 1st PCR result, positive COVID swab, latest COVID swab, admission, and 1st chest X-ray (CXR) were well reported, with 79-97% coverage, whilst dates of subsequent PCR tests/results, X-rays, ITU admission, intubation and death were present for just 4-50% of patients. Coverage for date of death increased from 14.6% to 66% when limiting analysis to the subset of patients for whom the death status had also been reported as positive.

Medical history

The presence of cardiovascular disease (CVS) and chronic kidney diseases (CKD) were both reported for approximately 90% of patients. The presence of other pre-existing conditions, hypertension, type 2 diabetes mellitus, and lung diseases were reported for 66%, 55% and 51% of patients, respectively. The use of angiotensin receptor blockers, ACE inhibitors (ACEI), and non-steroidal anti-inflammatory drugs (NSAID) were known for between 40-43% of patients. The patient's smoking status (never, previous, current) was known for 25% of patients, with the packs per year history known for 4.4%, increasing to 25% when filtering for patients with current or previous smoking status. Finally, the stage of chronic kidney disease (if CKD, stage) was available for 7.5% of patients overall, rising to 49% in the subset in which CKD is reported.

For all of these fields other than pack year history and CKD stage, the reporting includes the negative status of not having the condition. Missing values include that the presence of the condition was marked as unknown or left blank.

Admission metrics

Of the clinical measurements recorded when a patient is admitted to hospital, blood pressure (systolic and diastolic) was available for 84% of patients and was by far the most complete field in this category. The majority of remaining fields were reported for between 33-48% of patients. However, Ferritin, FiO2, Troponin I, Fibrinogen, and D-dimer were reported for 10-19% of patients, and Troponin T, APACHE score and O2 saturation for only 1-3% of patients.

COVID information

The most complete COVID information by far was the result of the 1st PCR test and death status, which were present for

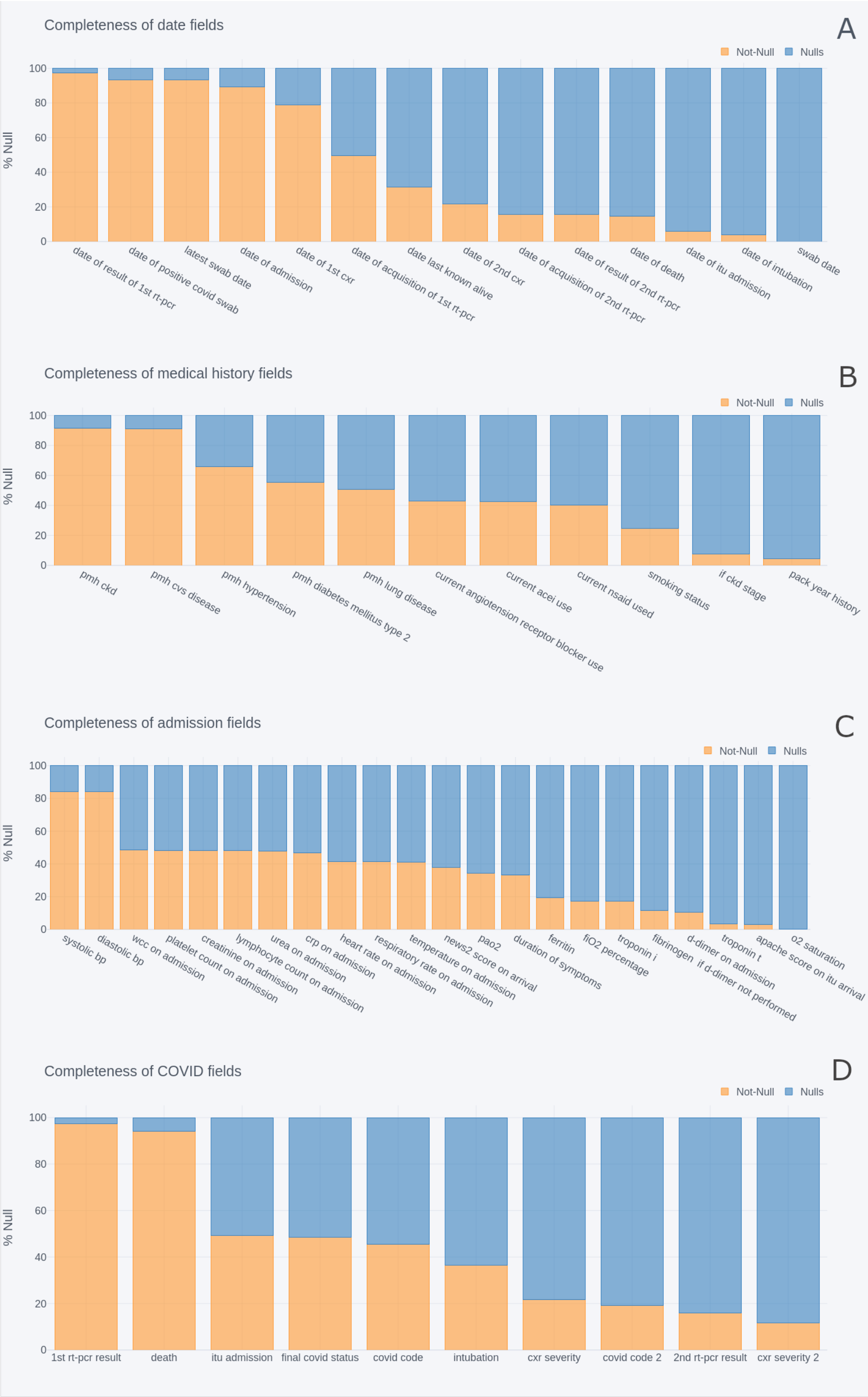

Figure 2. Completeness of clinical data fields related to (A) dates, (B) patient medical history, (C) symptoms on admissions and (D) COVID-related information.

97% and 94% of patients respectively. Admission to ITU, final COVID status and COVID code were reported for 45-49% of patients, and use of intubation for 36%. Beyond these the completeness of the fields declined, with chest X-ray severity data available for 21% of patients, COVID code 2 for 19%, result of second PCR test for 16% and chest X-ray severity 2 for 11%.

Image characteristics

This section is designed to inform users on general characteristics of the image data whilst also highlighting potential confounders that might hinder the ability to build effective AI models.

Subsequent sections of the analysis utilise the DICOM header tags associated with image files, these tags were read using open-source package Pydicom [12]. MRI images are excluded from all analyses due to low numbers in the database at the time of analysis.

Historic and acute

Both acute (related to COVID-19 hospital admission) and historic image studies (up to January 2017) are available for a subset of the NCCID patients. Historic image studies may be used to infer longitudinal risk factors or decouple the effects of pre-

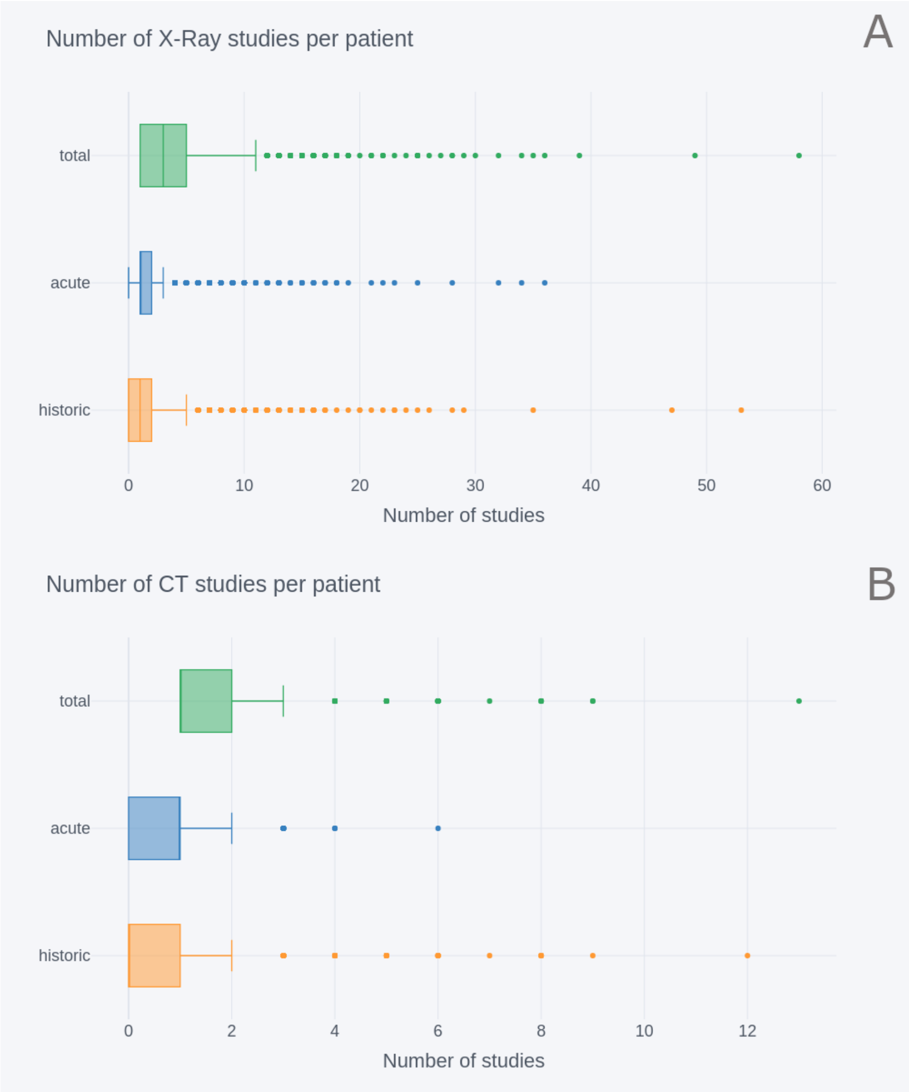

Figure 3. Number of historical/acute/total image studies per NCCID COVID-positive patient (n=2,826) for (A) X-rays and (B) CTs.

existing pathologies from COVID-related symptoms.

Figures 3 shows the distributions of the number of historical/acute/total X-ray (A) and CT (B) studies per COVID-positive patient. This number was calculated based on the date of admission and the DICOM *StudyDate* (*0008, 0020*), where a study was considered acute if it occurs on or after the admission date and historic otherwise. Date of admission was available through the clinical data for n=2,826 COVID-positive patients; reported results are based on this sample size. In both sets of boxplots, outliers are indicated by dots outside the limit of the plot whiskers and whiskers correspond to Q1 or Q3 +/- 1.5*iqr (interquartile range).

The total number of CTs per patient was median=1, iqr=1-2, this was lower than for X-rays (median=3, iqr=1-5). This consequently resulted in lower availability of acute CT studies, median=1, iqr=0-1, max=6, and even lower availability of historic CT studies, median=0, iqr=0-1, but with a handful of patients having 2-12 studies. For X-rays the median number of acute studies per patient was 1, similar to CT but the iqr=1-2 is higher, indicating that patients are more likely to have multiple X-rays taken in the acute setting. There was also more historic data available for X-rays, with a median=1, iqr=0-2.

Acquisition timing

The timing of imaging acquisition along the patient treatment pathway was investigated to understand if different modalities were used for differing purposes in the clinical setting. Two time lags were compared across X-ray studies and CT studies:

$$D_1 = date_{image} - date_{positiveSwabTaken} \quad (1)$$

$$D_2 = day_{image} - (date_{admission} - days_{durationOfSymptoms}) \quad (2)$$

Image dates were established from the *StudyDate* field of the DICOM headers and lags were calculated based on the first image after the admission date of each patient. This limited analysis to the images taken during the patient's treatment for COVID-19 in the acute setting. Box plots are used because of the skewed nature of timing data. The distributions of these lags are shown for X-ray (orange) and CT (blue) scans in Figure 4 A and B.

For A), the median offset between swab date and study date was -1 day for X-rays and +1 day for CT scans. The high number of -1 day lags for X-ray shows that the majority of X-rays had been taken before a patient's COVID-19 status was known. The overall distribution across X-rays was far narrower, with an

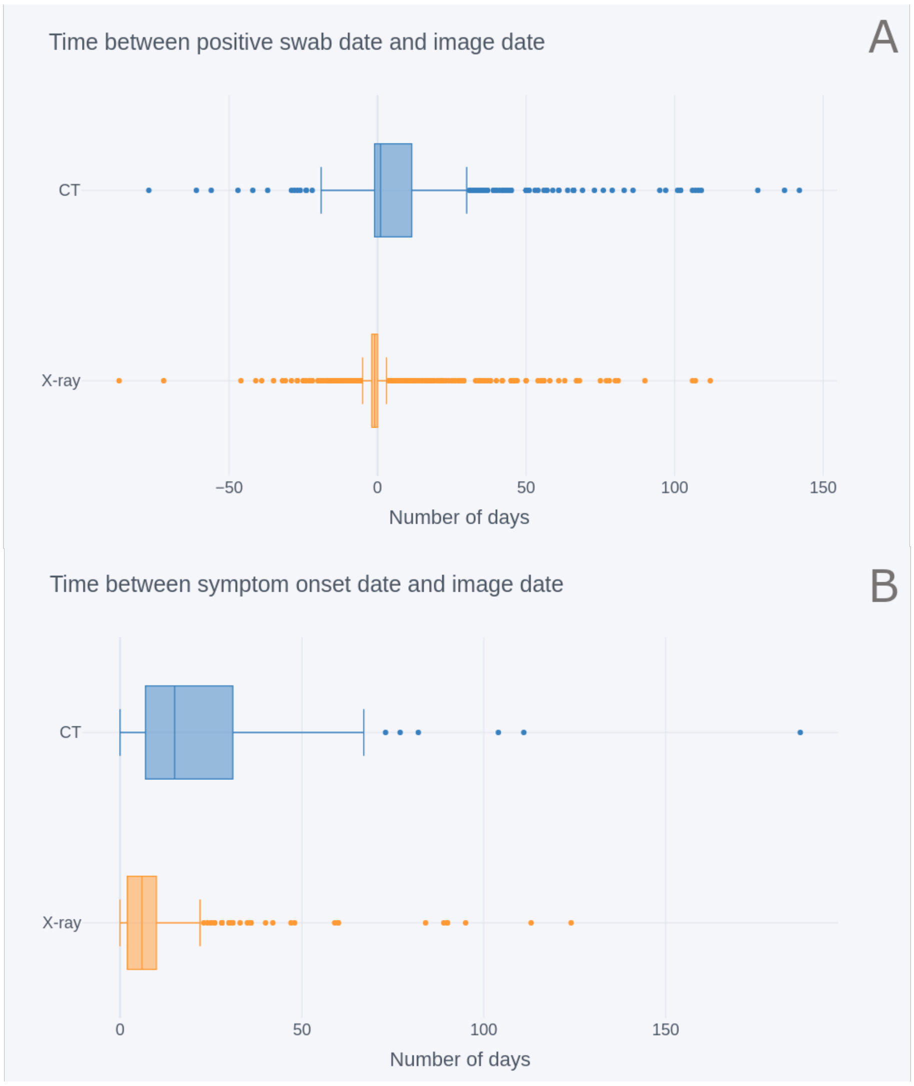

Figure 4. (A) Number of days between the patient's RT-PCR swab test and the image acquisition (n_{XRAY} = 2,410, n_{CT} = 507) and (B) Number of days between patient symptom onset and image acquisition (n_{XRAY} = 803, n_{CT} = 133)

iqr= -2-0 compared to iqr= -1-12 for CTs. This suggests that the timing of X-rays is very consistent across patients, whereas longer tails in the CT distribution indicates more variance of usage between patients.

Both modalities display outliers with large negative offsets. These negative offsets suggest that some patients had images taken up to 87 days prior to the positive RT-PCR swab. In practice, the majority of these cases are likely driven by data quality issues surrounding ambiguous dates, such as 03/10 vs 10/03.

The delay between onset of symptoms and image dates tell a similar story to the above. X-rays had a median offset of 7 days (iqr = 3-11 days), whilst CTs had a median offset of 15 days and a wider iqr = 8 - 34 days. Although calculated on a smaller subset of studies (936 compared to 2917) for which duration of symptoms data was available, this analysis corroborates the hypothesis that X-rays were consistently used earlier in the care pathway, potentially as diagnostic aids.

Scanner Types

To investigate the variety of medical imaging equipment within the NCCID database, two analyses were performed:

- Study counts by machine manufacturer were generated using the *Manufacturer* attribute (0008, 0070) from the DICOM headers.
- Study counts for model types available within each manufacturer were generated through the combination of DICOM attributes *Manufacturer* + *Manufacturer's Model Name* (0008, 1090). This combined attribute is hereby referred to as model. The results for this additional breakdown are provided in Appendix B.

In both cases, all available DICOM tags were read from each X-ray image file in a study, but only from the first file of each CT study, as the DICOM attributes of interest were the same across all files in a given CT study. Studies for the positive cohort were filtered to exclude historical data based on *DICOM Acquisition Date* (0008, 0022) and date of admission.

Manufacturers

The counts of scanner manufacturers across NCCID positive (orange) and control (blue) cohorts are displayed in Figure 5, where ordering of manufacturers is based on the total counts (positive+negative). The total, non-historic, study counts across all manufacturers were 11,086 (*positive* = 5552, *negative* = 5534) for X-ray and 1746 (*positive* = 634, *negative* = 1112) for CT.

The largest suppliers for X-rays were Fujifilm, Siemens and Philips Medical Systems, which contributed 2687, 2588 and 2297 studies each. The next largest supplier was Carestream

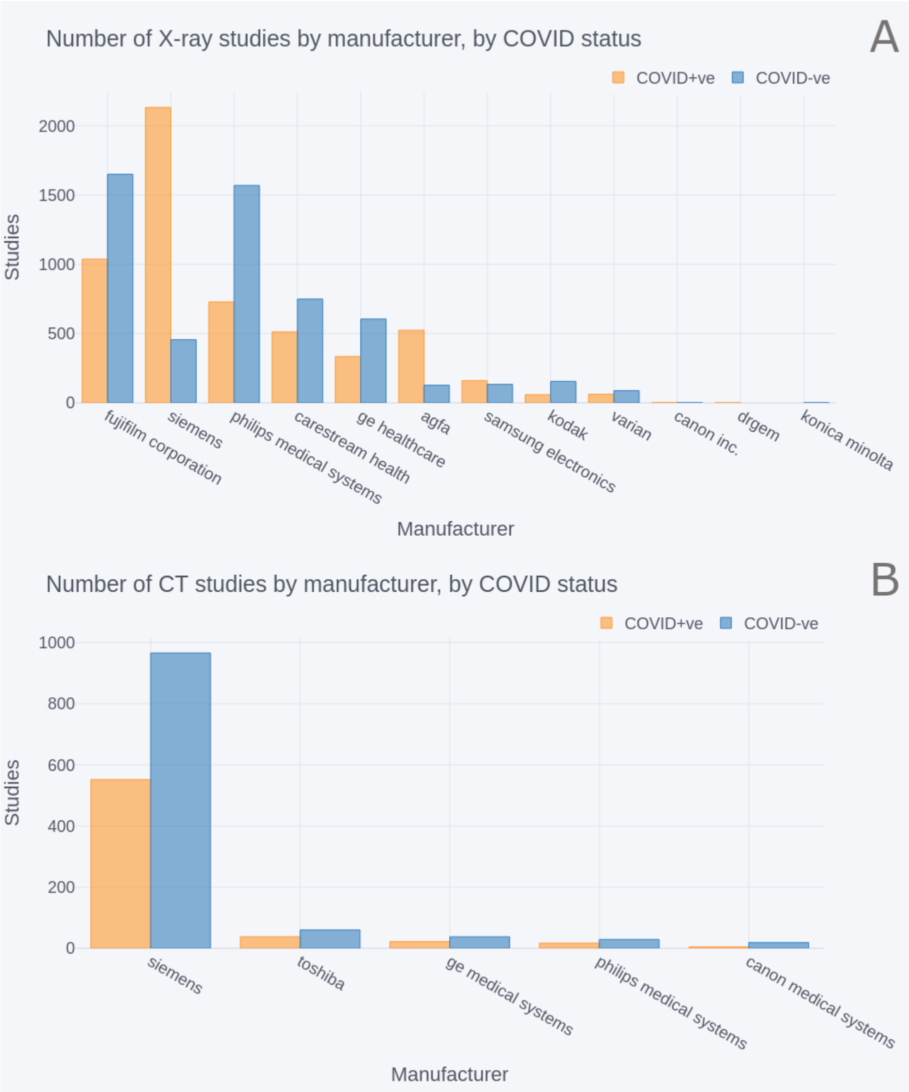

Figure 5. Number of COVID-positive and negative (A) X-ray studies by manufacturer and (B) CT studies by manufacturer. In both cases the manufacturers are ordered by highest to lowest total (positive+negative) number of studies

Health, with 1261 studies, after which the number of studies steadily declined for the remaining 8 suppliers. In the case of CT studies, Siemens far outweighed the other 4 providers, accounting for 1518 studies.

All X-ray and CT manufacturers had studies for both positive and negative patients. However, some manufacturers, such as Siemens, had significantly more studies in one of the two groups.

Portable versus stationary

It was suspected that X-ray data in the NCCID originates from a combination of portable and stationary machines. This was partly a consequence of operational restrictions caused by the pandemic, where portable scanners were easier to regularly disinfect and could be transported to dedicated COVID-19 wards as part of infection control procedures [2]. As such, the use of portable machines was expected to be more prevalent in the COVID-positive cohort of the NCCID.

The percentage of portable scanners was estimated to investigate the presence of potential model confounders caused by e.g., lower image resolution in portable scanners:

- Studies with references to portable, e.g., *CHEST PORTABLE* in the *Body Part Examined* attribute (0018, 0015) were counted. Different variations were mapped e.g., *PORT CHEST* to *CHEST PORTABLE*. Studies that did not include any reference to portable in this attribute were assumed to originate from stationary scanners.
- Counts were then adjusted by taking the unique set of eight models from the above step (highlighted in Table ?? of the Appendix) and extrapolating the portable status to all studies acquired on these models, under the assumption that operators forgot to indicate portability in these cases.

Table 3 displays estimated portable machine counts within the NCCID training data, excluding historic images. For positive patients, there were 78 studies labelled with some reference to portable in their Body Part Examined DICOM attribute (original counts), accounting for approximately 1.4% of X-ray studies. In comparison, the number of portable machines indicated by this DICOM attribute accounted for 0.9% of negative patient studies. After extrapolating the portable status to all studies taken on the models where portability was indicated at least once, the proportion of X-ray studies taken on portable devices increased to approximately 14.3% for positive patients and 16.7% for negatives (adjusted counts).

Table 3. Estimated number of X-ray studies originating from either stationary or portable machines for COVID positive and negative patients.

Scanner type	COVID-positive		COVID-negative	
	original count	adjusted count	original count	adjusted count
**stationary**	5489 (98.6%)	4770 (85.7%)	5490 (99.1%)	4610 (83.3%)
**portable**	78 (1.4%)	795 (14.3%)	49 (0.9%)	927 (16.7%)

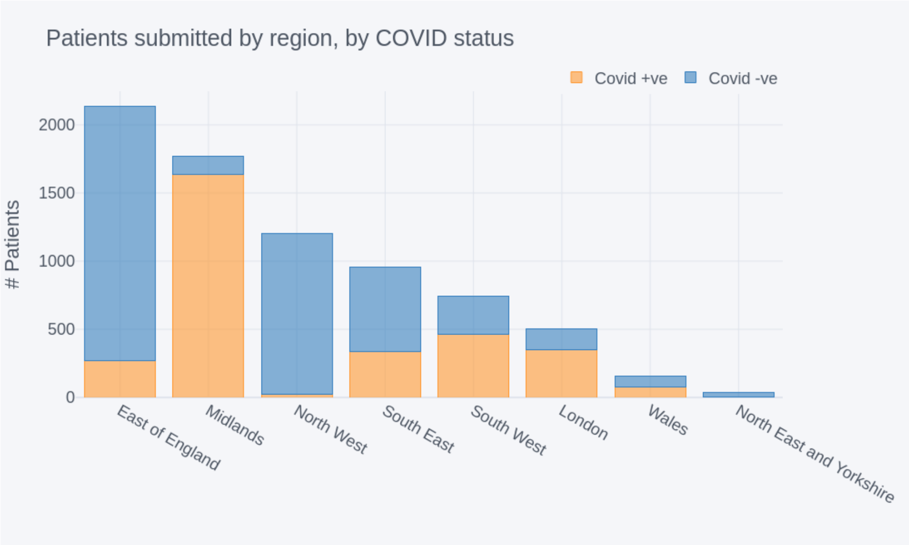

Figure 6. NCCID positive and negative patients submitted by region, sorted by total contribution.

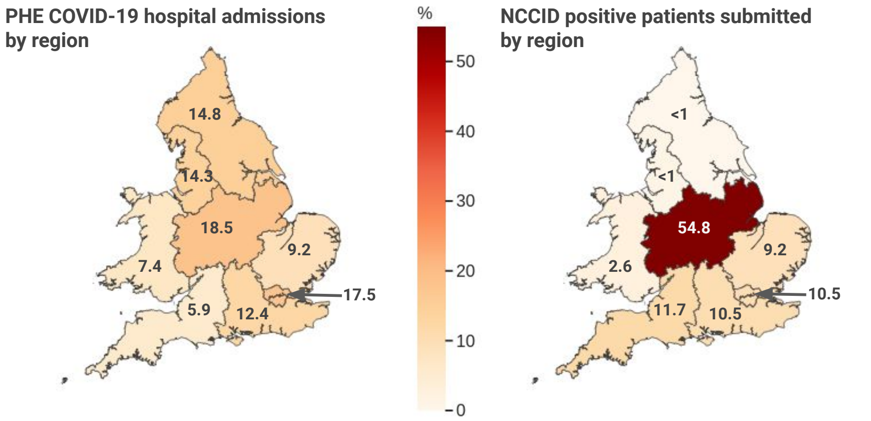

Figure 7. Comparison of national COVID-19 admissions at a regional level with NCCID positive cases.

Cohort Analysis

This section explores the geographic, demographic and temporal coverage of the NCCID database. The aim is to measure if/how the NCCID differs from the general COVID-affected population and how any disparities might limit the generalisability of AI solutions.

Geographic Coverage

Figure 6 details the number of patients submitted to the NCCID from each NHS England region [13] and Wales, split by their confirmed COVID-19 status, as measured via a RT-PCR swab test (positive = orange, negative = blue). The regional data were aggregated from the 19 sites that had submitted data by the analysis cut-off date.

In addition, Figure 7 displays two choropleth maps showing (A) the proportion of COVID-19 hospital admissions, within each NHS England region and Wales, as reported by Public Health England [14] and (B) the proportion of COVID-19 positive patients in the NCCID for the same geographic boundaries. Boundary data was sourced from the ONS geoportal [15].

The highest proportion of data originated from the East of England region, which accounted for 2,134 patients in total. However, the vast majority of these (1,862) were negative patients, submitted by a single site. The second highest reporting region was the Midlands, with a combined total of 1,769 patients in the database. In contrast to the East of England, the vast majority of patients submitted in the Midlands were positive cases (1,638), and 1,511 of these originated from a single site.

Other regions submitted less data overall, but regions in the South of England (including London) and Wales had comparatively even contributions of positive and negative cases. Coverage of positive cases in the North of England and Yorkshire

was limited, with the North East and Yorkshire region having only 33 patients in total.

The NCCID's geographic coverage of COVID-19 patients was largely concentrated in the Midlands, accounting for 54.8% of positive patients in the training data. After the Midlands, the East of England, London, South East and South West of England accounted for 41.6% of positive patients in total (9.2%, 10.2%, 10.5%, and 11.7%, respectively). Data from Wales, the North West, and the North East and Yorkshire regions collectively made up just 3.6% of NCCID positive patients.

This was at odds with COVID-19 hospital admissions (as reported by PHE) which were more evenly spread across England and Wales. Specifically, London, the Midlands, North East and Yorkshire and the North West accounted for approximately 15-18% of admissions each. Wales, the South East, East of England and South West accounted for smaller proportions of 10.3%, 9.8%, 7.0% and 5.1% of admissions, respectively.

Demographic Coverage

The purpose of this section is to establish how generally representative the NCCID cohort is of the population hospitalised due to COVID-19 and whether good representation carries through to the most severe outcomes (through the mortality variable). Understanding the underlying causes of any demographic differences in COVID-19 prevalence or outcomes is beyond the scope of this paper.

Subsequent to applying the cleaning and merging pipeline (see clinical data methods section), demographic data was available for sex=85%, ethnicity=69%, and age=86% of patients in the NCCID (n=3,168). Distributions of these categories within the NCCID were compared against reference datasets, where available, or COVID-related statistics reported by the International Severe Acute Respiratory and Emerging Infection Consortium (ISARIC) [16, 17] and the general UK population reported by the 2011 national census. Equivalent comparative data was not publicly available for Wales, as such, data from Welsh health boards is excluded from the subsequent demographic results. Comparisons were made for both admissions and mortality rates where the total sample size of patients with recorded deaths was n=694. In all subsequent comparison plots the NCCID is indicated using blue and comparative datasets are displayed in orange and green.

The NCCID is a subsample of the population that is hospitalised due to COVID-19, and a dynamic resource that will continue to grow over the coming months. It is sensible to assume that the sample of NCCID data being scrutinised in this paper will deviate from the final population of both the NCCID and general COVID-effected population. To account for some of this sampling error in the below comparisons, we applied a bootstrap method to generate confidence intervals for the NCCID data. The plotted proportions of a given category, e.g., percentage of patients aged 18-64, represent the median percentage across 1000 bootstrap samples. Similarly, error bars on the subsequent plots represent the 95% confidence interval (ci) of measurements across the bootstrap samples. In each case, the sample size of the bootstrapped distributions was equal to the size of the relevant original NCCID sample (i.e., if the original NCCID sample had n=3000 patients with sex data available then the bootstrapped samples each contained n=3000 entries).

Sex

Figure 8A compares the split of male ($n = 1,797$) and female ($n = 1,295$) positive cases within the NCCID to that of the general UK population via the 2011 national census [18] $n = 63,182,000$, and the COVID-effected population reported by ISARIC [16], $n = 20,113$. At 58% male to 42% female (ci = 56-60%male:40-44%female), the NCCID was more closely aligned to the 60:40 ratio reported in COVID-19 admissions than the 51:49 split of the general UK population.

Figure 8B compares the male:female mortality rates within the NCCID cohort (n=673) against those reported by NHSE (n=32,483), up to the cut-off date, 29/10/2020 [19]. The NHSE mortality data exhibited a male to female ratio of 61:39. This fell within the 95% confidence interval for the NCCID, 60-67%:33-40%.

Ethnicity

Figure 9A compares the ethnicity proportions (Asian, Black, Other, White) of NCCID patients, n=2854, against the general UK population as reported in the 2011 UK census, n=63,182,000, [18] and the COVID-affected population reported by ISARIC, n=30,693 [17].

The White group accounted for 83% of individuals in both the census and ISARIC populations. In contrast, only 72% (ci = 70-73%) of NCCID COVID-positive patients were from White ethnic backgrounds. This was counterbalanced by higher proportions of Asian (median=14%, ci=13-16%) and Black (median=9%, ci=8-10%) people, than observed in either the Census (Asian = 9%, Black = 3%) or ISARIC (Asian = 5%, Black = 4%). In addition, ISARIC reported higher proportions of patients from Other minority backgrounds (8%) than in NCCID (median=5%, ci=4-6%), whilst the census data indicated that approximately 4% of the UK population belonged to this group.

Figure 9B compares the ethnicity proportions within the subset of NCCID patients that have recorded deaths and ethnicity data (n=633) to the ethnicity proportions reported by NHSE for COVID-19 in-hospital deaths in England [19], up to the reporting cut-off date (n=29,610).

Similar to the admissions data above, the NCCID mortality data was under-representative of the White ethnic group (median=78% ci=74-81%), and over-representative of the Asian (median=11%, ci=9-13%) and Black (median=8%, ci=6-10%) groups, compared to mortality rates in the broader COVID-population (White=85%, Asian=8%, Black=5%).

Age

Figure 10 compares the percentage of NCCID patients within a set of age bands (0-5, 6-17, 18-64, 65-85, 85+) to the percentages for COVID-19 hospital admissions across England, as reported by Public Health England [14]. The comparisons are shown at both the national level as well as within each NHS England region.

As reflected in the geographic analysis, regions in the North of England had insufficient data to make meaningful comparisons. Specifically, data availability was below the suppression threshold in all age groups for the North East and Yorkshire and most age groups for the North West. The error bars for the remaining age groups in the North West, 18-64, and 65-85, spanned 30-34 percentage points respectively.

Amongst the regions that had enough data to support comparisons, most showed no statistically significant differences between the NCCID and PHE. For London (n_{PHE} = 25,804, n_{NCCID} = 353) and the South East (n_{PHE} = 15,690, n_{NCCID} = 335) PHE data fell within the NCCID confidence intervals for all age-groups. The two data sets were closely aligned in the South West (n_{PHE}= 26,876, n_{NCCID}= 463), where only the 18-64 and 65-85 age bands fell outside the confidence interval by just 1% each. Similarly, in the East of England (n_{PHE}= 11,252, n_{NCCID}= 272), the PHE data for the 18-64 age group was again just 1% outside the upper bound for the NCCID, and all other age bands fell within the confidence interval.

The single exception was the Midlands, which exhibited a large difference of 18% (ci=15-20%) between PHE (n=26,661) records and the NCCID (n=1638) for the 18-64 age band. This was counterbalanced by smaller proportions of over 65s than observed by PHE. These deviations can be reasonably attributed

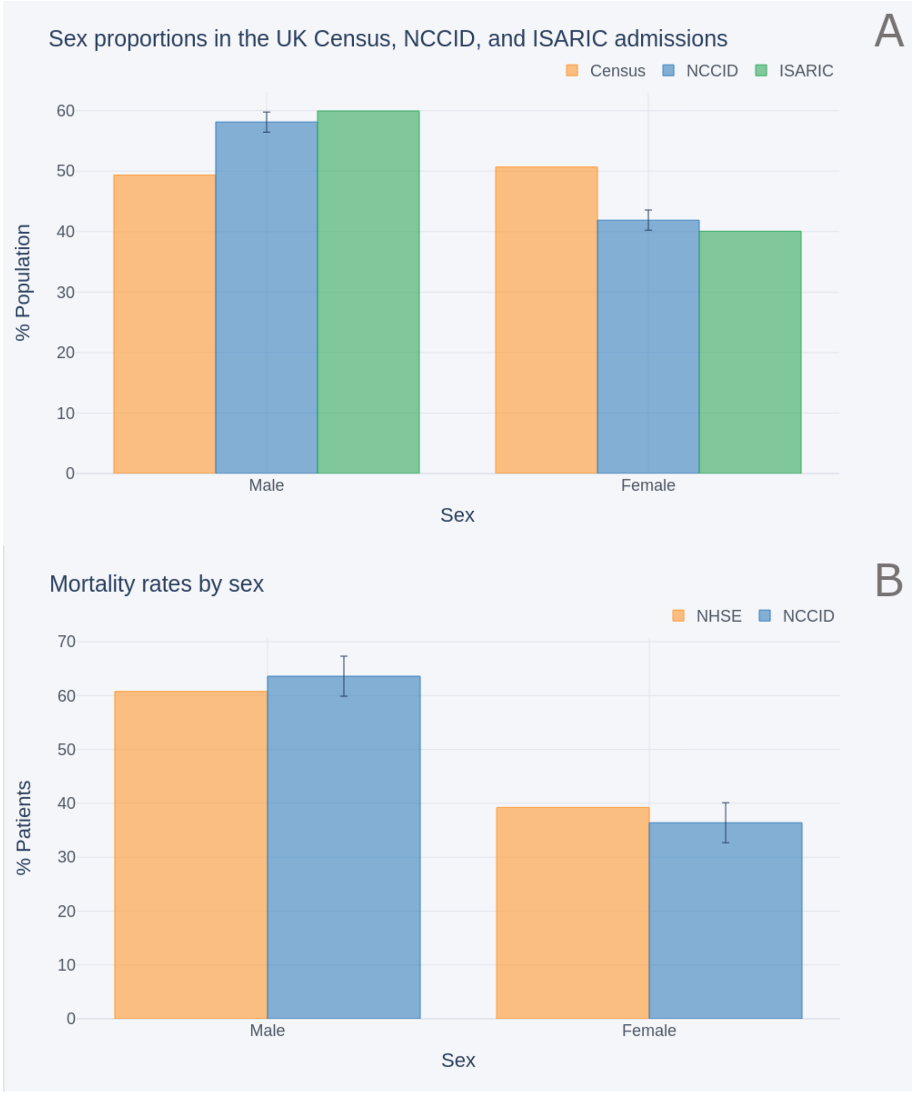

Figure 8. Comparison of sex split within: (A) the NCCID COVID-19 patients, the general UK population (as reported in the 2011 census) and COVID-19 hospital admissions (reported by ISARIC); (B) NCCID recorded deaths and NHS England COVID-19 hospital mortality data.

to the fact that data was collected by a single site, located in an urban area. Furthermore, given that the Midlands contributed a substantial volume of positive patients to the NCCID, this overrepresentation of 18-64 year olds extended to the national level comparison ($median_{NCCID}$= 42%, ci = 40-43%, n_{NCCID}= 3088, $median_{PHE}$= 33.7%, n_{PHE}= 137,757).

The NCCID had low numbers of patients in the 0-5 group at a national level, and low numbers for the 6-17 group in all geographies.

Figure 11 compares age breakdown of NCCID patients with recorded deaths to age breakdowns of in-hospital COVID-related deaths reported by NHSE [19]. A different set of age bands were used to align to the NHSE data: 0 - 19, 20 - 39, 40 - 59, 60- 79, 80+.

Although the age bands used by NHSE (n=32,484) are different to those used in the admissions comparisons above, we can see a general knock-on effect, where over-representation of younger people in the dataset resulted in a larger percentage of 40-59 year olds with recorded deaths in the NCCID (median=10%, ci=8-13%, NHSE=7%).

Temporal Coverage

This section investigates the approximate hospital admission dates of the NCCID patients to identify how well the NCCID has captured patients across the course of the pandemic. The total number of NCCID patients with a positive RT-PCR swab test occurring each week since 1 March 2020 was compared to the total number of confirmed COVID-19 patients admitted to hospital each week for the same period according to PHE data [14]. This analysis was performed at a national level, including data across the whole of England and Wales. Given that there were (at the time of study) no NCCID sites in Scotland and Northern Ireland, data from these nations was omitted from PHE admissions calculations. The two time-series are displayed in Figure 12.

The peak of both datasets was aligned, occurring on 5 April, with a gradual decrease in numbers until the summer period, July to September 2020. From September onwards the national COVID-19 admissions began to rise again, however this was not (up to the analysis cut-off 29/10/20) reflected by a rise in positive patients admitted into the NCCID database.

Re-use Potential

Findings of data completeness analysis

Clinical information is an important complement to the chest images. Gaps in the clinical information can deprive re-

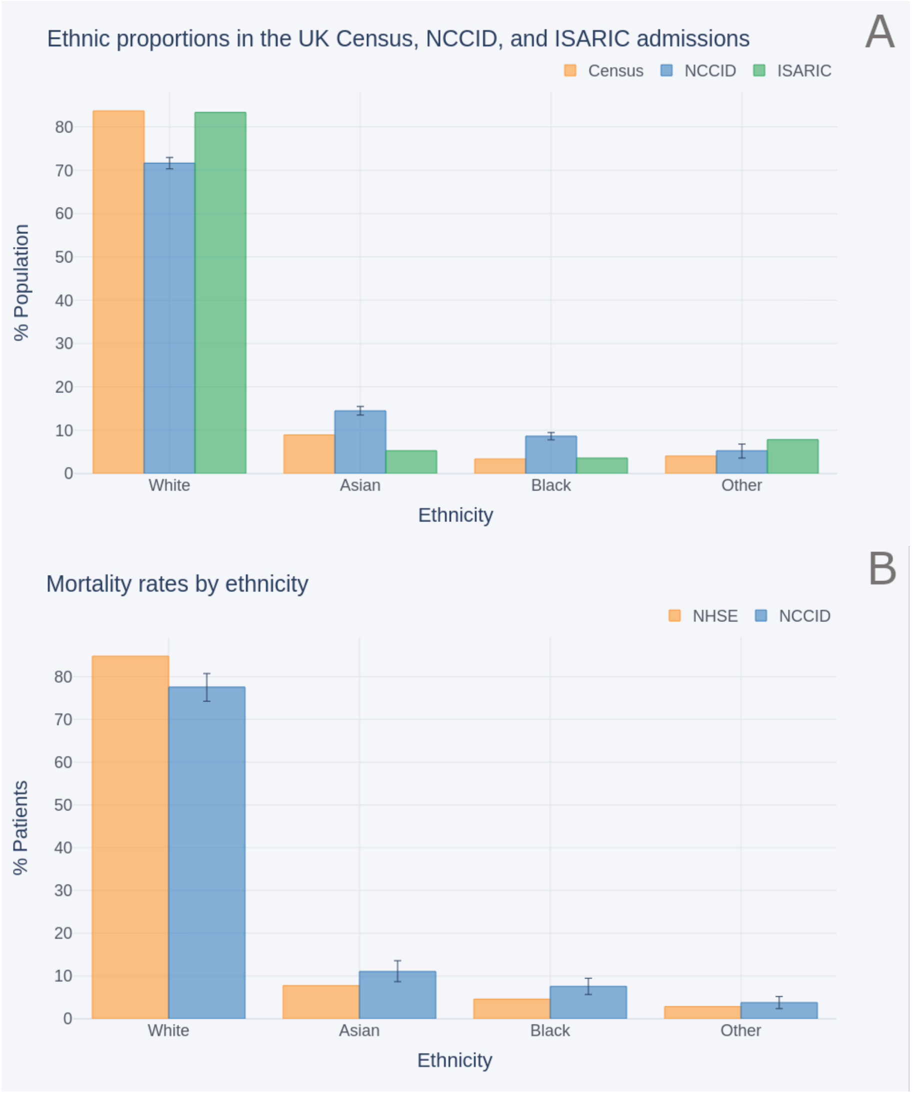

Figure 9. Comparison of ethnicity proportions within (A) the NCCID COVID-19 patients, the UK population (as reported in the 2011 national census) and COVID-19 hospital admissions (reported by ISARIC); (B) the NCCID recorded deaths and NHS England COVID-19 hospital mortality data.

searchers of contextual data on the patient's health for inclusion in analyses and ML models. For instance, incompleteness of the *FiO2* data may hinder the development of mortality or deterioration risk scores that take this field into account. Analogously, since clinical information may be used to control for confounders, missing entries can reduce a researcher's ability to draw firm conclusions from the data.

The overall availability of clinical data varies by each field in the dataset. Key dates including when the RT-PCR swab was taken and when a patient was admitted to hospital are well covered, and can provide useful insight into the timelines of image acquisition during the patient care pathway (e.g., Figure 4).

The occurrence of pre-existing conditions is also relatively well characterised, particularly for cardiovascular and kidney diseases. This information should allow data users to account for the effects of comorbidities in their analyses, which have been shown to play a significant role in disease outcomes for COVID-19 patients [20, 21, 22, 23].

Information relating to the patients' conditions upon hospital admission (e.g., blood pressure and white-cell count) were the least well reported, with a mean of 65% null values in this category compared to 49% for dates, 53% for medical history, and 56% for COVID-19 fields. Data users should also be aware that the reporting units for these metrics may vary between sites, making it difficult to disambiguate overlapping values, and causing artificially high variances for some metrics (Appendix C). To remedy this, we plan to make site-specific unit information available to users once collated, even though it is unlikely that all participating sites will be able to provide such information. It should also be noted that some of the missing data originates from the fact that specific hospitals do not commonly measure all of the listed metrics. For example, several sites report that they do not routinely measure *Troponin T* on admission. Furthermore, some fields such as O2 saturation are obsolete and no longer requested in the data collection spreadsheet.

Overall, the causes of missing information in the NCCID are difficult to identify because of their number and diversity. It is nevertheless known that the following factors have contributed to incompleteness of clinical data across the different categories:

- Staff at data-collection sites may have been unable to fill in certain fields due to time pressure and the emergency situation.
- Depending on the site, data has been gathered by staff (research nurses, radiologists, etc.,) with access to different

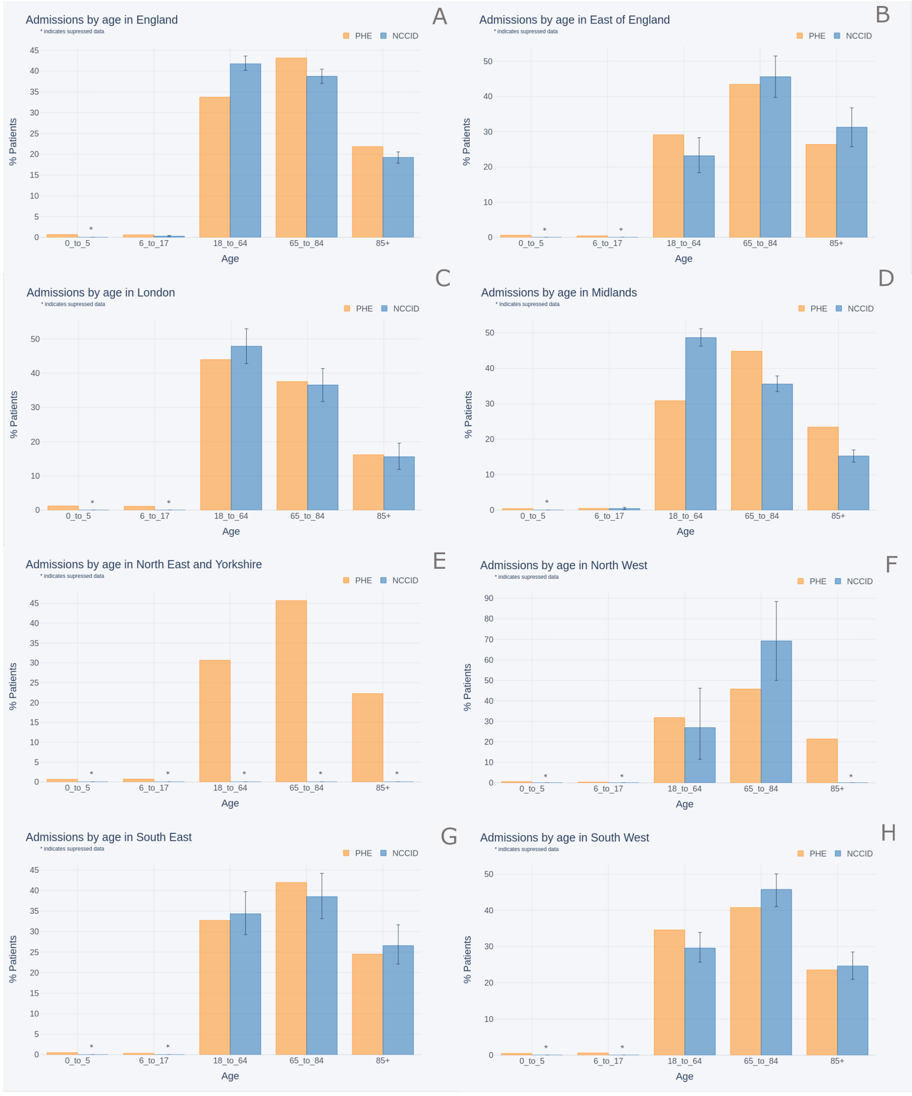

Figure 10. Comparison of age proportions between COVID-19 hospital admissions (reported by PHE) and NCCID positive patients for (A) England, (B) East of England, (C) London, (D) Midlands, (E) North East and Yorkshire, (F) North West (G) South East and (H) South West.

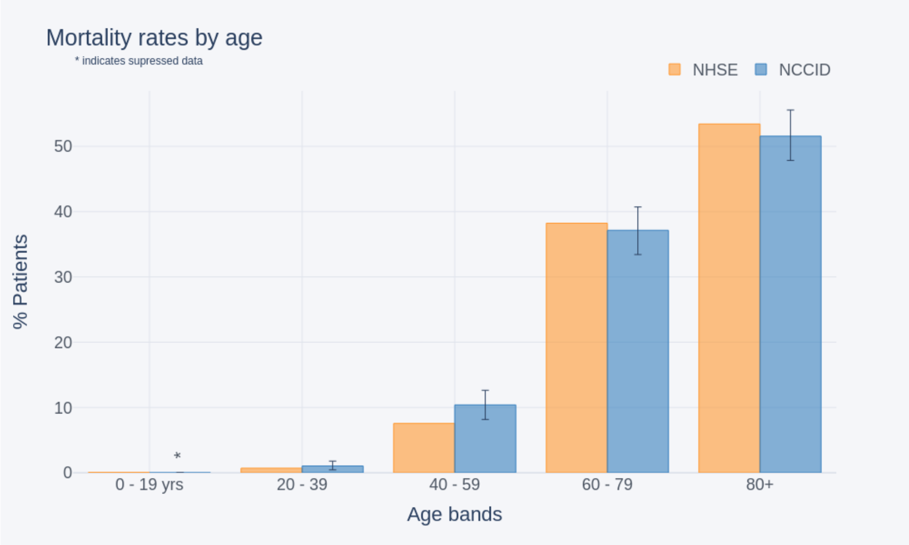

Figure 11. Comparison of age distributions between recorded COVID-19 deaths (as reported by NHSE) and the NCCID (England only).

clinical information systems and records. Therefore, the person collecting and uploading data to the NCCID may have been unable to get hold of specific clinical information.

- Certain fields could only be present in a relevant subset of

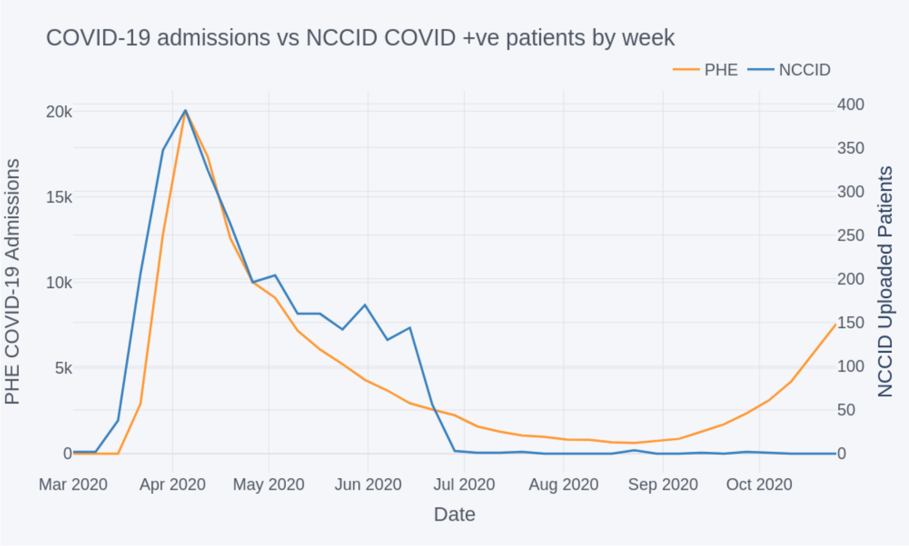

Figure 12. Comparison of COVID-19 admissions to NCCID positive cases by week.

patients, and were otherwise left empty. For example, a few fields referred to secondary RT-PCR swab tests (*date of acquisition, date of result, result*) and secondary chest X-rays (*date, severity*), which were only required, and consequently filled in for some patients. Additionally, the reporting of date of death, and stage of chronic kidney disease were much higher when selecting the subset of patients for whom death or presence of kidney disease had been reported. Similar effects are likely to be the underlying cause of the relatively high occurrence of missing values in COVID-19 fields such as *ITU admission*, *intubation* and *severity of disease in secondary images*[11].
- Information such as medical history may not have been provided by the patient, for example because they were incapacitated.
- Data may not have been gathered as part of routine clinical practice, see the above remarks.

Plans are in place to establish a link between the NCCID and ISARIC-4C [24] that will automatically populate clinical information for patients included in both datasets. This link aims to improve the availability of clinical data in the NCCID whilst relieving the burden on clinical staff to provide additional information.

Findings of image characteristics analysis

Historic and acute

The number of total, acute or historic image studies varied across COVID-positive patients. In general, patients were less likely to have historic CT data available (median=0 studies), compared to X-ray (median=1 study). This is likely driven by the general disparities in availability between the two modalities, given that X-rays are faster and cheaper to acquire, and are therefore more frequently used in the UK clinical setting. Investigators that wish to incorporate historical data as a means of accounting for pre-existing pathologies or understanding longitudinal risk factors should possibly focus on X-ray studies.

Both X-ray and CT had a median of 1 study per patient, but there were many more X-ray studies available overall (approximately 12,000 compared to 1,500). It is sensible that researchers building diagnostic tools should focus on X-ray data, as these are also likely to be most useful in the UK clinical setting. However, given that CTs are likely to be used in the more severe/difficult cases, those wishing to analyse disease severity/prognosis can utilise CT data. One advantage of the CT data is that it provides much richer imaging information, encoded into a 3D volume where different view planes and slices through the relevant anatomy can be probed. In comparison, X-ray image resolution tends to be higher but only a single projection is possible.

The total number of MRI studies is currently too low (17 studies) to be useful in the machine learning setting. This is likely to remain true even as the database grows, as low numbers are caused by the rarer adoption of MRI in the treatment of COVID-19 patients, which in turn, limits the clinical relevance of this modality.

Acquisition Timing

Analysis of image timings with respect to patient PCR-RT swab dates and onset of symptom dates revealed that X-rays were predominantly used at the early stages of a patient's care pathway. Interestingly we identified the median offset between swab date and X-ray was -1 day, which suggests that X-rays were commonly being used as diagnostic aids. This is likely a result of limited testing capacity during the earlier stages of the pandemic. In contrast, CT images were generally used later in the care pathway, with greater variance between patients on the specific timing of scans. These findings reflect BSTI clinical guidelines for the UK, which stipulated that CT should be used sparingly as a diagnostic tool, to preserve capacity for normal operation [11].

Concentrating on the response to COVID-19 in the UK and the NCCID, data users may want to focus on building diagnostic tools using X-ray images, and could potentially use CT scans to study disease severity, progression and prognosis. It remains to be seen whether improved testing capacity or other factors will modify the timings for either modality in the later stages of the pandemic, and therefore change the technological needs of the response to COVID-19 in the UK.

Scanner types

X-ray and CT images present in the NCCID were captured on a range of systems from multiple manufacturers, providing variability in the type of images available. This was true for both positive and negative patients, although the ratio of positive to negative varied somewhat by manufacturer. Users of NCCID should take into account the relative frequencies of imaging across the different manufacturers (and models) to minimise unwanted bias. For instance, Siemens is the dom-

inant manufacturer for CT, but large amounts of X-ray data was available for a number of providers, which could help produce generalisable models.

Due to limitations imposed by the pandemic, it was suspected that imaging data in the NCCID would originate from a combination of portable and stationary X-ray machines. Portable machines are easier to quickly sanitise between sessions and could more readily be moved to quarantine wards as part of hospital infection control measures, making it possible that there would be a higher prevalence of such machines in the patient cohort [2]. Exploration of the DICOM headers initially identified a small proportion of positive scans (1.4%) acquired on portable devices, with just over half of this this percentage negative scans (0.9%). This was then extended to all studies taken on the same scanner models, such that 14.3% of positive X-rays and 16.7% of negative X-rays were estimated to come from portable machines. These preliminary findings do not suggest a large imbalance in the ratio of portable and non-portable scanners between the positive and control cohorts. However, in lieu of a more definitive method for identifying portable machines from DICOM information we estimated prevalence based on notes in the Body Part Examined attribute. It is plausible that this method under-estimates the true number of portable scanners, as such, further investigation of this issue is recommended. Examining a sample of images from the various devices may provide a more robust measure of portability for data users but the above analysis serves to highlight this aspect of the NCCID data.

Awareness of potential model confounders is crucial to ensure efficacy of ML models, particularly with respect to how performance generalises beyond the training data. For instance, significant disparities in the prevalence of certain equipment types between the positive and control cohorts could produce an ML model that successfully differentiates the two groups. However, is it conceivable that the decision boundaries in such a model are based on attributes of the medical imaging machinery (e.g., resolution, projection etc.) rather than disease related attributes [25]. Data users should take care to balance their training samples, ensuring a good variety of scanner types within both cohorts, to build models that generalise well to the variety of clinical imaging equipment used in the UK. Indeed, there are many additional confounders to be aware of including but not limited to (see Appendix B):

- Digital radiography (DR) vs computed radiography (CR) which are different techniques for digitising the X-ray signal, either directly from the panel (DR) or by scanning cassette-based phosphor storage plates into digital format (CR).
- Photometric interpretation, which refers to the image contrast such that MONOCHROME1 scans should be inverted to match MONOCHROME2 scans or vice versa.
- View positions, e.g., Anterior-Posterior (AP), Posterior-Anterior (PA), Lateral (LL), etc.

By collecting data from multiple Trusts and Health Boards across the UK, the NCCID strives to provide a training database that can cover many of these confounding factors, and improve the efficacy of any resulting machine learning models in the clinical setting.

Findings of cohort analysis

Geographic Coverage

At time of analysis, the NCCID was not evenly sampled across the participating regions. We observed that COVID-19 positive-patients in the database largely originated from the Midlands, and very few patients originated from Wales and Northern England (Figure 6).

Several factors may underpin these disparities, including: 1) the number of NCCID sites within each region 2) the size and population coverage at each hospital site; 3) the number of positive COVID-19 cases recorded at each site; 4) the duration of time the site has been contributing to the NCCID for; and 5) the availability of research coordinators and PACS teams to upload all cases. Reason 3, is unlikely to be the driving factor, as indicated by Figure 7 in which PHE reported a more equal distribution of COVID-19 hospital admissions.

Low submissions from the North of England reflect the relatively small number of participating NCCID sites in these regions. The fact that the uptake of the programme has been uneven across different regions can be attributed to factors such as the reach of our professional network, constrained availability of staff to support our database, and variable responsiveness of local sites to national initiatives.

Regional disparities in the number of positive and negative cases submitted are more likely to be driven by factor 5, the capacity of PACS teams. The guidance given to hospital sites was to submit all positive cases with images taken in the acute setting, and a smaller sample of negative cases with acute imaging (approximately 100 per week if available). Due to the request for accompanying clinical data in positive cases, it is much easier for sites to submit negative cases, for whom only the images and a small number of clinical data points are required.

Demographic Coverage

The NCCID aims to be a UK-wide initiative assembling a database that is as representative as possible of the entire population. Nevertheless, the present geographical coverage of the NCCID is partially skewed, which, if additional data curation is not applied rigorously, may produce biases in ML models trained on this resource. For example, issues may occur because of the incorrect representation of specific demographic groups and clinical risk factors such as pre-existing conditions [16, 26]. Indeed, we observed some of these downstream effects in the population analysis, particularly in the regional proportions of age-groups within the NCCID, which deviated most significantly from PHE data in the Midlands and Northern England. These effects accumulated in a general over-representation of younger adult patients compared to more elderly patients in the NCCID for both admissions and mortality.

In addition, the NCCID contains very low numbers of patients in the 0-5 and 6-17 age groups, partly because of the active omission of under-11s due to small counts, where the underlying cause is the low prevalence of symptomatic COVID-19 in children [27, 28]. Reduced availability of data for under-18s limits the use of the NCCID to adult diagnostic/prognostic models for the time being. This may change as the database grows, particularly as the exclusion of data from under-11s will be stopped once sufficiently high numbers are available.

The ethnic composition of the NCCID deviated from the 2011 UK census data. Whilst establishing the causes of this discrepancy would require additional investigation, the over-representation of Asian and Black groups for the admission data may, to some extent, be due to differences in the incidence of COVID-19. As a matter of fact, several studies have indicated higher corrected hospitalisation odds ratios for minority ethnic groups compared to people of white backgrounds [29, 30, 17, 16]. The reliability of the comparison between the NCCID and the census, however, is diminished by the fact that the latter is a decade old, so that more recent estimates (including the imminent 2021 national census) could exhibit a significant demographic shift in the benchmark for the UK population as a whole.

The comparison with ISARIC data was crucial for understanding how representative the NCCID is of the COVID-19 pa-

tient population that it is sampled from. Again, the NCCID displayed higher percentages of Asian and Black patients and lower percentages of White patients than the hospital admissions data from ISARIC. A similar effect was seen in the comparison with mortality data from NHSE.

The reasons why the NCCID diverges from other datasets in relation to ethnicity are not fully understood. Nevertheless, we believe that the most likely issue is the uneven geographical representation of the NCCID. This would be consistent with the fact that the Asian and Black groups are overrepresented, and the White group is underrepresented in every comparison of the NCCID with other nationwide datasets (UK census, NHSE and ISARIC). It is clear from the literature that the distribution of ethnicities in COVID related hospital admissions varies considerably between different regions [14, 26]. For example, Sapey et al. [31], which looked specifically at COVID positive hospital admissions from around Birmingham saw a much higher proportion (18.5%) of patients of South Asian ethnicity. Apea et al. [32], which carried out a similar analysis looking at COVID positive hospital admissions from around East London, saw a much higher proportion of patients of both South Asian and Black ethnicity (31% and 20% respectively). In an analogous way, the fact that a large fraction of the data in the NCCID has been collected in an urban area of the Midlands may have increased the representation of Asian and Black groups, and reduced that of the White group.

The male to female ratio of NCCID patients was found to closely align with the 60:40 split reported for COVID-patients by ISARIC. This is a departure from the approximately 50:50 split expected in the general population, as measured by the 2011 census data (where sex ratios are less likely to significantly vary over time, making the age of the census less of a limiting factor), and reflects findings of other COVID-19 studies [33, 34, 23]. A similar increased hazard ratio was observed in the male to female mortality rates, where the NCCID was well aligned to NHSE in hospital deaths data. Data users should be aware that there is a class imbalance (as is common in clinical studies) but unlikely to be severe enough to prevent the training of models that will generalise.

Overall, data users should keep in mind that, owing to the variable incidence of COVID-19, the NCCID is expected to have slightly different demographic composition to the general population. Several studies have reported different COVID-19 prevalence rates between men and women, ethnic groups and age groups [35, 16, 23, 33, 34, 17, 31, 30]. As more sites are on-boarded and the database grows, we expect the composition of the NCCID to more closely reflect the populations reported by e.g., PHE, ISARIC, and NHSE. For the meantime, data users should be aware of these differences, and how underrepresentation of certain groups might affect model performance for those individuals. Whilst the risk of model unfairness relating to demographic disparities is less obvious in medical imaging than for other ML applications (e.g., facial recognition for law enforcement [36]), it is probable that disease manifestation differs across age groups and ethnicities, or that the prevalence of comorbidities varies across ethnicities and between urban and non-urban populations. Therefore, these characteristics may still have negative effects on the fairness of ML models. Furthermore, disease-related class imbalances play a relevant role in quantifying algorithmic bias, where fairness definitions based on pure demographic parity [37, 38] may provide misleading measures of success and failure in this problem space, unless corrected to the relevant ratios.

Temporal Coverage

The low numbers of positive cases uploaded to the NCCID training dataset since September 2020 suggest that the data capture pipelines were (up to the analysis cut-off in October) still processing the large backlog of patients from the first wave of the pandemic. Users should note that ML models built from the training data will capture the characteristics of the first peak, and may not generalise completely to patients admitted during the subsequent winter peaks, particularly in view of the emergence of a new strain of SARS-CoV-2, lineage B.1.1.7 [39]. Failures to generalise over time could arise from several factors, including:

- potential changes to disease manifestation associated with the new strain of SARS-CoV-2 that has dominated prevalence in the UK starting from December 2020 [40, 41], though such effects are speculative at the time of publishing;
- the prevalence of flu-related comorbidities, expected to be more common in winter months;
- any changes in the use of imaging for diagnostic/prognostic purposes between the early stages and later stages of the pandemic;
- changes to treatment policies over time (such as the introduction of dexamethasone) and how these affect disease severity;
- the roll-out of the COVID-19 vaccination programme, which in the UK has begun on 8 December 2020 [42], and has delivered almost 18 million first doses [14] at the time of writing;
- changes to non-pharmaceutical interventions (behavioural restrictions like lockdowns) and the down-stream effects these have on which members of the population are exposed to the virus.

It is noteworthy that COVID-19 admissions for the general population peaked at approximately 20,000 per week (for the period and regions studied in this article), whilst the peak of positive patients in the NCCID was orders of magnitude lower, at just under 400. Any statistics or models derived from the NCCID database are therefore likely to suffer from sampling error, which should be considered when reporting such analyses.

Next Steps

The NCCID has made significant progress within the space of a few months to collect a sizable dataset to support research into COVID-19. However, there are a number of next steps, summarised below, which the NCCID initiative aims to implement in the short-to-medium term in order to better support data users:

i. We will re-engage with existing hospital sites to understand the reasons behind a decline in submission of recent cases and implement mitigating actions (see point 5).
ii. We will engage new sites across the UK, focusing on rural and other underrepresented geographies, such as the North of England, Wales, Northern Ireland (point iv) and Scotland (point iii) to expand the geographic and demographic coverage of the NCCID.
iii. We will implement a linkage with the Scottish National PACS and Safe Haven Network.
iv. In Northern Ireland we will start by establishing a linkage with the Northern Trust PACS team.
v. We will implement a connection with the ISARIC-4C [24] dataset to improve the completeness of the clinical data fields while reducing the burden on hospital staff, since the data is linked across as opposed to collected afresh. It is hoped that lighter data-gathering processes will attract new sites, and motivate existing ones to contribute even more to the database.
vi. We will carry out investigative work beyond clinical variables and metadata into the quality of the images themselves

so as to assess their utility for algorithmic development.

vii. We will implement automation pilots in a selection of sites to establish a continuous feed of images for positive and negative patients. Clinical data for these sites will be provided through the ISARIC-4C linkage.

Conclusion

This paper aimed to provide further detail on the content of the NCCID's training dataset, in order to support existing data users with their research efforts, raise awareness for the NCCID as a valuable resource that others may want to access, and inform both existing and potential data users of improvements we aim to make in future. The decision to publish this paper now, rather than after the improvements have been made, reflects the iterative nature of this particular initiative, and the urgency presented by the pandemic to ensure information is made available as quickly, transparently and securely as possible. The NCCID initiative has collected a large volume of imaging and clinical data within a short period of time; this has been achieved through the expertise of NCCID partners, lean agile delivery methods, and the prioritisation of COVID-19 response work. However, there are a number of considerations in the NCCID training dataset to be aware of, namely: 1) the limitations of its geographic and, consequently, demographic representation; 2) issues with clinical data quality and completeness. We have identified a number of improvements to address these considerations, and will continue to expand and refine the quality of the NCCID training dataset. Despite these limitations the NCCID provides a valuable resource to the medical imaging community, addressing many of the common pitfalls highlighted in a recent meta-analysis of COVID-19 imaging models [25]. In particular, as a centralised resource, housing high quality DICOM imaging data and clinical attributes for thousands of patients, across a variety of imaging machinery, the NCCID is large enough to mitigate many of the data quality/bias concerns of smaller fragmented resources, making it an important tool in supporting the response to the COVID-19 pandemic.

Data Availability

The NCCID training data is available to any users, including software vendors, academics and clinicians, via a rigorous Data Access Request (DAR) process. Applications are adjudicated by an independent committee based on several factors including but not limited to relevance to COVID-19 and compliance with information governance regulations. The required paperwork and additional instructions are detailed on the website.

Availability of source code

The codebase for the data warehouse is open source and available through the NHSX github:

- Project: covid-chest-imaging-database
- Operating system(s): e.g. Platform independent
- Programming language: Python
- License: MIT

The open-source data ingestion and cleaning pipeline can be found on NHSX github:

- Project: nccid-cleaning
- Operating system(s): e.g. Platform independent
- Programming language: Python
- License: MIT

Availability of supporting materials

Additional information on the NCCID, including an overview of participating sites, existing data processors, live updates on the size of the training data and instructions for requesting access are all available through the main webpage.

More information on guidelines and data schemas for the clinical data are available through RSNFT, further detail is also provided through the HDRUK portal.

Additional Files

Appendix

Declarations

Ethical Approval and Consent for publication

The legal basis for the NCCID is provided by the notice under regulation 3(4) of the UK National Health Service (Control of Patient Information) Regulations 2002 (COPI Notice), and ethical approval was obtained for the NCCID to operate as a research database by the UK Health Research Authority. The initiative has received Ethics approval by both the Health Research Authority (HRA) and the Scottish Public Benefit Privacy Panel (PBPP). As the NCCID only contains pseudonymised information, individual consent to publish is not required.

Competing Interests

No conflicts of interest to declare.

Funding

The NCCID is publicly funded by NHSX. Joseph Jacob was supported by a Wellcome Trust Clinical Research Career Development Fellowship (209553/Z/17/Z) and by the NIHR BRC at UCL.

Author's Contributions

D.C. provided supervision, project administration and support on funding acquisition. Ot.B., S.D, F.L, E.J, provided project administration and supported the reviewing and editing of the manuscript. Os.B and R.B contributed to the literature review and sections of the manuscript, in addition R.B provided project administration, and helped conceptualise the analysis. T.G. performed/supervised the data analysis, drafted the manuscript, contributed to software and helped conceptualise the analysis. D.S. performed parts of the data analysis and contributed to the manuscript, helped conceptualise the analysis and contributed to software. A.C. helped conceptualise/support parts of the data analysis and contributed to software. G.I. provided conceptual input, implemented the data warehouse and contributed to software, parts of the data analysis and manuscript. J.J and A.F provided project supervision, conceptual input, project administration and reviewed/edited the manuscript. M.H-B. provided conceptual input, implemented the data collection infrastructure, contributed to software, project administration, and other resources. J.C.W provided conceptual input and reviewed/edited the manuscript. The NCCID collective is responsible for curating and providing the data at participating hospital sites.

Acknowledgements

The authors would like to thank the following individuals for their contributions to this work: Ayub Bhayat, Hena Aziz, Zain Eisa, Rob Howieson, Alison Lowe, Aliya Rafique, Anastasios Sarellas and Giuseppe Sollazzo.

References

1. Hosseiny M, Kooraki S, Gholamrezanezhad A, Reddy S, Myers L. Radiology perspective of coronavirus disease 2019 (COVID-19): lessons from severe acute respiratory syndrome and Middle East respiratory syndrome. American Journal of Roentgenology 2020;214(5):1078–1082.
2. Kooraki S, Hosseiny M, Myers L, Gholamrezanezhad A. Coronavirus (COVID-19) outbreak: what the department of radiology should know. Journal of the American college of radiology 2020;17(4):447–451.
3. Shi H, Han X, Jiang N, Cao Y, Alwalid O, Gu J, et al. Radiological findings from 81 patients with COVID-19 pneumonia in Wuhan, China: a descriptive study. The Lancet infectious diseases 2020;20(4):425–434.
4. Lee EY, Ng MY, Khong PL. COVID-19 pneumonia: what has CT taught us? The Lancet Infectious Diseases 2020;20(4):384–385.
5. Chung M, Bernheim A, Mei X, Zhang N, Huang M, Zeng X, et al. CT imaging features of 2019 novel coronavirus (2019-nCoV). Radiology 2020;295(1):202–207.
6. Kanne JP, Chest CT findings in 2019 novel coronavirus (2019-nCoV) infections from Wuhan, China: key points for the radiologist. Radiological Society of North America; 2020.
7. Cleverley J, Piper J, Jones MM. The role of chest radiography in confirming covid-19 pneumonia. bmj 2020;370.
8. NHSX AI Lab;. Accessed: 2021-03-23. https://www.nhsx.nhs.uk/ai-lab/about-nhs-ai-lab/.
9. Jacob J, Alexander D, Baillie JK, Berka R, Bertolli O, Blackwood J, et al. Using imaging to combat a pandemic: rationale for developing the UK National COVID-19 Chest Imaging Database. European Respiratory Journal 2020;56(2).
10. Watson J, Whiting PF, Brush JE. Interpreting a covid-19 test result. Bmj 2020;369.
11. BSTI: Thoracic Imaging in COVID-19 Infection.;. Accessed: 2021-03-23. https://www.bsti.org.uk/media/resources/files/BSTI_COVID-19_Radiology_Guidance_version_2_16.03.20.pdf.
12. Pydicom;. Accessed: 2021-03-23. https://pydicom.github.io/.
13. NHS Regional Teams;. Accessed: 2021-03-23. https://www.england.nhs.uk/about/regional-area-teams/.
14. Public Health England: coronavirus dashboard;. Accessed: 2021-03-23. https://coronavirus.data.gov.uk/.
15. ONS geography portal: NHS England Regions (April 2020) Boundaries EN BFE;. Accessed: 2021-03-23. https://geoportal.statistics.gov.uk/datasets/nhs-england-regions-april-2020-boundaries-en-bfe.
16. Docherty AB, Harrison EM, Green CA, Hardwick HE, Pius R, Norman L, et al. Features of 20 133 UK patients in hospital with covid-19 using the ISARIC WHO Clinical Characterisation Protocol: prospective observational cohort study. bmj 2020;369.
17. Harrison EM, Docherty AB, Barr B, Buchan I, Carson G, Drake TM, et al. Ethnicity and outcomes from COVID-19: the ISARIC CCP-UK prospective observational cohort study of hospitalised patients 2020;.
18. 2011 Census: Population Estimates for the United Kingdom, March 2011;. Accessed: 2021-03-23. https://www.ons.gov.uk/peoplepopulationandcommunity/populationandmigration/populationestimates/bulletins/2011censuspopulationestimatesfortheunitedkingdom/2012-12-17.
19. NHS COVID-19 Daily Deaths;. Accessed: 2021-03-23. https://www.england.nhs.uk/statistics/statistical-work-areas/covid-19-daily-deaths/.
20. Guan Wj, Liang Wh, Zhao Y, Liang Hr, Chen Zs, Li Ym, et al. Comorbidity and its impact on 1590 patients with COVID-19 in China: a nationwide analysis. European Respiratory Journal 2020;55(5).
21. Wang B, Li R, Lu Z, Huang Y. Does comorbidity increase the risk of patients with COVID-19: evidence from meta-analysis. Aging (Albany NY) 2020;12(7):6049.
22. de Lucena TMC, da Silva Santos AF, de Lima BR, de Albuquerque Borborema ME, de Azevêdo Silva J. Mechanism of inflammatory response in associated comorbidities in COVID-19. Diabetes & Metabolic Syndrome: Clinical Research & Reviews 2020;14(4):597–600.
23. Petrilli CM, Jones SA, Yang J, Rajagopalan H, O'Donnell L, Chernyak Y, et al. Factors associated with hospital admission and critical illness among 5279 people with coronavirus disease 2019 in New York City: prospective cohort study. Bmj 2020;369.
24. ISARIC 4c;. Accessed: 2021-03-23. https://isaric4c.net/.
25. Roberts M, Driggs D, Thorpe M, Gilbey J, Yeung M, Ursprung S, et al. Common pitfalls and recommendations for using machine learning to detect and prognosticate for COVID-19 using chest radiographs and CT scans. Nature Machine Intelligence 2021;3(3):199–217.
26. Pollán M, Pérez-Gómez B, Pastor-Barriuso R, Oteo J, Hernán MA, Pérez-Olmeda M, et al. Prevalence of SARS-CoV-2 in Spain (ENE-COVID): a nationwide, population-based seroepidemiological study. The Lancet 2020;396(10250):535–544.
27. Ludvigsson JF. Systematic review of COVID-19 in children shows milder cases and a better prognosis than adults. Acta paediatrica 2020;109(6):1088–1095.
28. Dong Y, Mo X, Hu Y, Qi X, Jiang F, Jiang Z, et al. Epidemiology of COVID-19 among children in China. Pediatrics 2020;145(6).
29. Martin CA, Jenkins DR, Minhas JS, Gray LJ, Tang J, Williams C, et al. Socio-demographic heterogeneity in the prevalence of COVID-19 during lockdown is associated with ethnicity and household size: Results from an observational cohort study. EClinicalMedicine 2020;25:100466.
30. Sze S, Pan D, Nevill CR, Gray LJ, Martin CA, Nazareth J, et al. Ethnicity and clinical outcomes in COVID-19: a systematic Review and Meta-analysis. EClinicalMedicine 2020;p. 100630.
31. Sapey E, Gallier S, Mainey C, Nightingale P, McNulty D, Crothers H, et al. Ethnicity and risk of death in patients hospitalised for COVID-19 infection in the UK: an observational cohort study in an urban catchment area. BMJ open respiratory research 2020;7(1):e000644.
32. Apea VJ, Wan YI, Dhairyawan R, Puthucheary ZA, Pearse RM, Orkin CM, et al. Ethnicity and outcomes in patients hospitalised with COVID-19 infection in East London: an observational cohort study. BMJ open 2021;11(1):e042140.
33. Gebhard C, Regitz-Zagrosek V, Neuhauser HK, Morgan R, Klein SL. Impact of sex and gender on COVID-19 outcomes in Europe. Biology of sex differences 2020;11:1–13.
34. Klein SL, Dhakal S, Ursin RL, Deshpande S, Sandberg K, Mauvais-Jarvis F. Biological sex impacts COVID-19 outcomes. PLoS pathogens 2020;16(6):e1008570.
35. Public Health England: Disparities in the risk and outcomes of COVID-19;. Accessed: 2021-03-23. https://assets.publishing.service.gov.uk/government/

uploads/system/uploads/attachment_data/file/908434/Disparities_in_the_risk_and_outcomes_of_COVID_August_2020_update.pdf.
36. Independent report on the London Metropolitan Police Service's trial of live facial recognition technology.;. Accessed: 2021-03-23. http://repository.essex.ac.uk/24946/1/.
37. Begley T, Schwedes T, Frye C, Feige I. Explainability for fair machine learning. arXiv preprint arXiv:201007389 2020;.
38. Mehrabi N, Morstatter F, Saxena N, Lerman K, Galstyan A. A survey on bias and fairness in machine learning. arXiv preprint arXiv:190809635 2019;.
39. Rambaut A. et. al, Preliminary genomic characterisation of an emergent SARS-CoV-2 lineage in the UK defined by a novel set of spike mutations;. Accessed: 2021-03-23. https:https://virological.org/t/.
40. Kirby T. New variant of SARS-CoV-2 in UK causes surge of COVID-19. The Lancet Respiratory Medicine 2021;9(2):e20–e21.
41. Volz E, Mishra S, Chand M, Barrett JC, Johnson R, Geidelberg L, et al. Transmission of SARS-CoV-2 Lineage B. 1.1. 7 in England: Insights from linking epidemiological and genetic data. medRxiv 2021;p. 2020–12.
42. BBC: First COVID vaccine UK.;. Accessed: 2021-03-23. https://www.bbc.co.uk/news/uk-55227325.

manuscript figure

Click here to access/download;Figure;fig1-nccid-ifra5.png

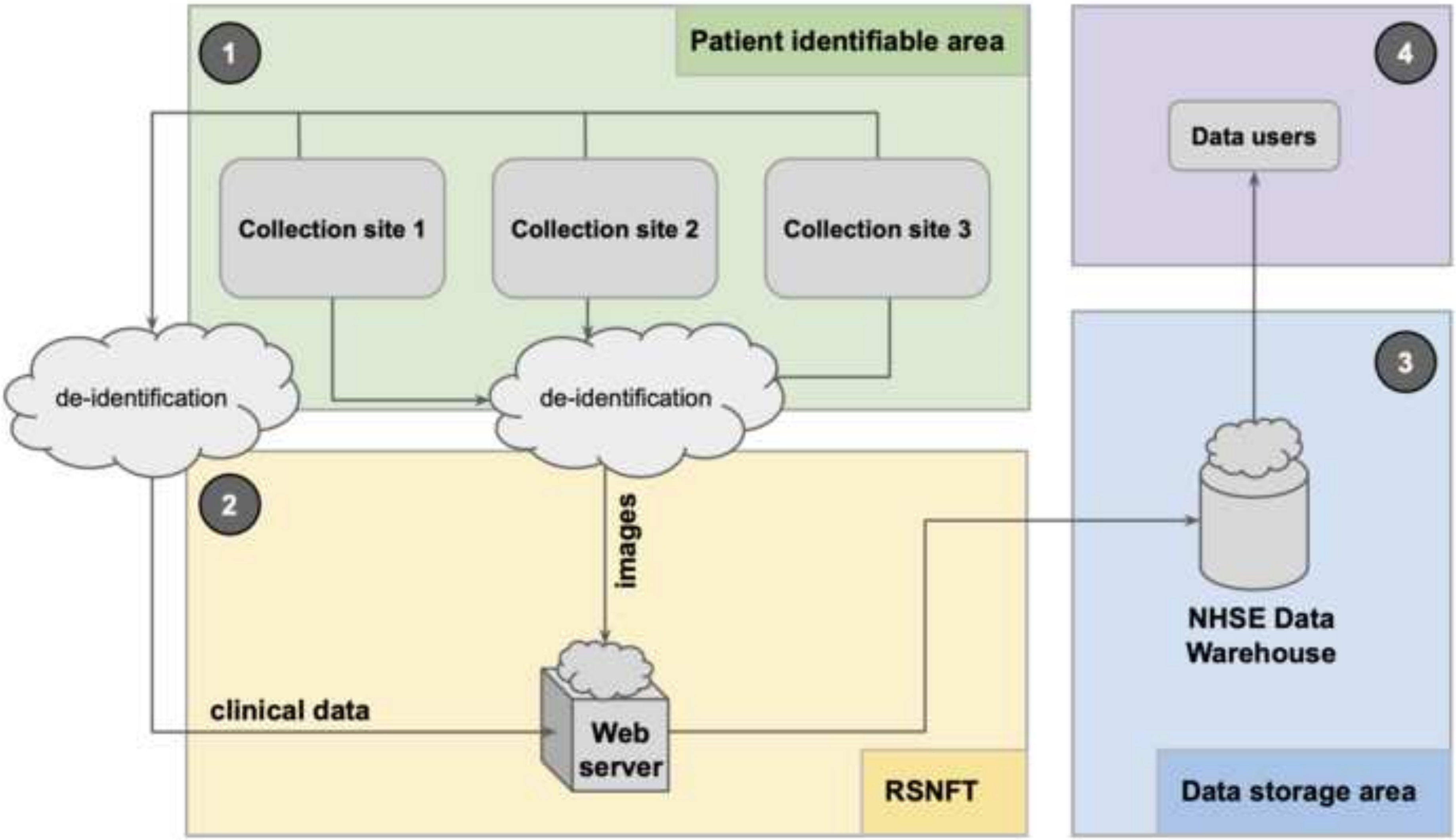

manuscript figure

Click here to access/download;Figure;fig2-completeness.png

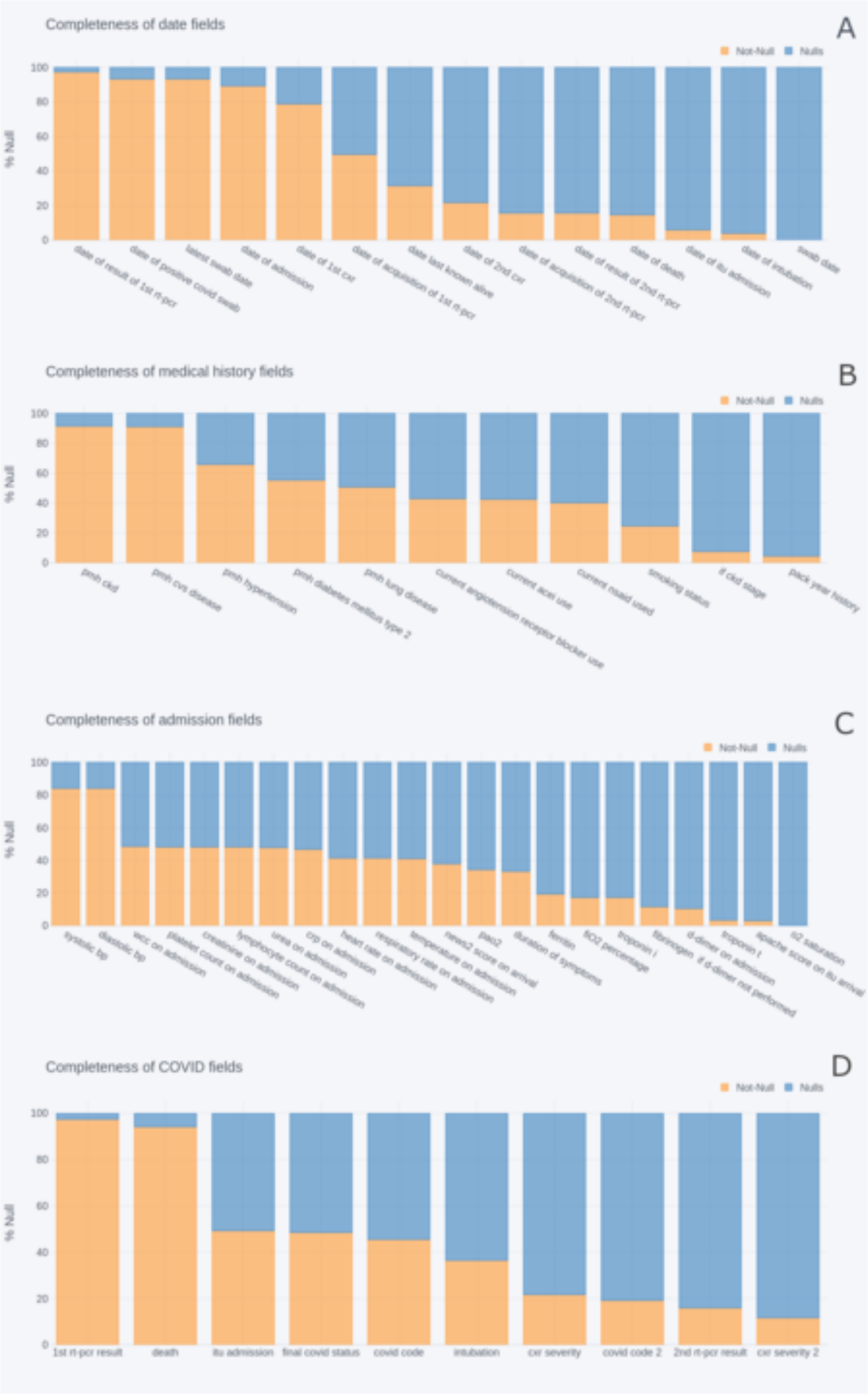

manuscript figure Click here to access/download;Figure;fig3-historic.png

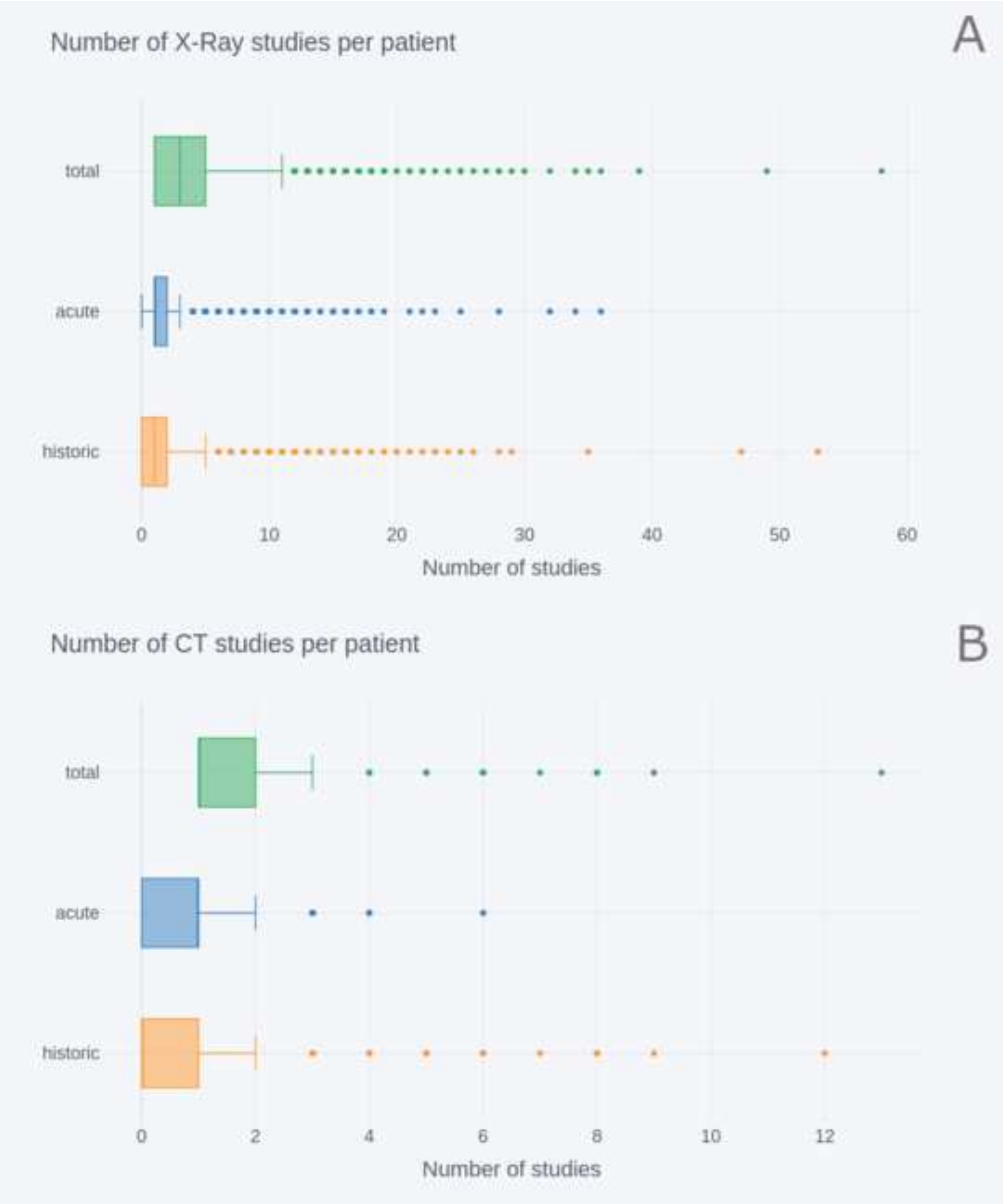

manuscript figure Click here to access/download;Figure;fig4-imagetiming.png

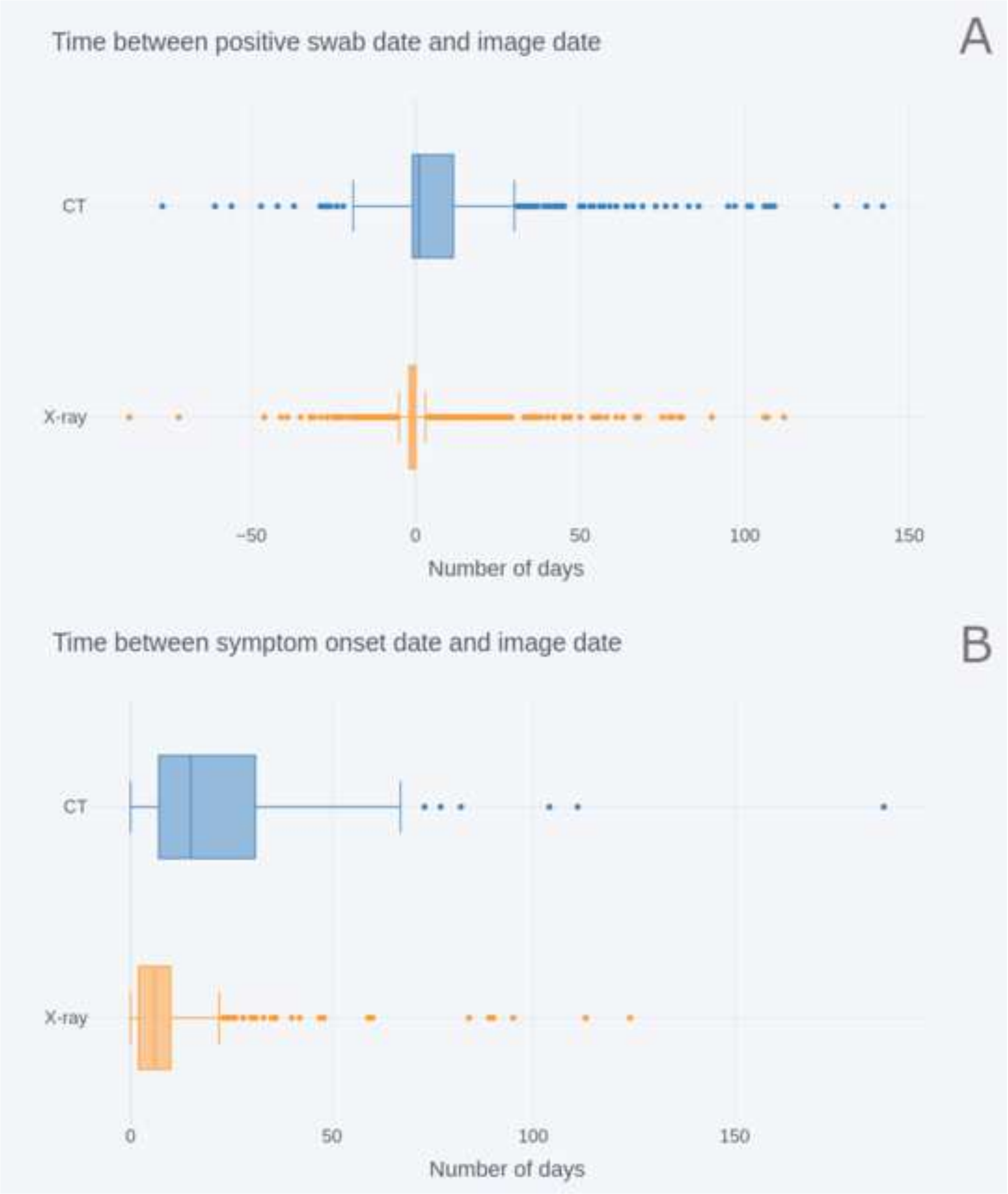

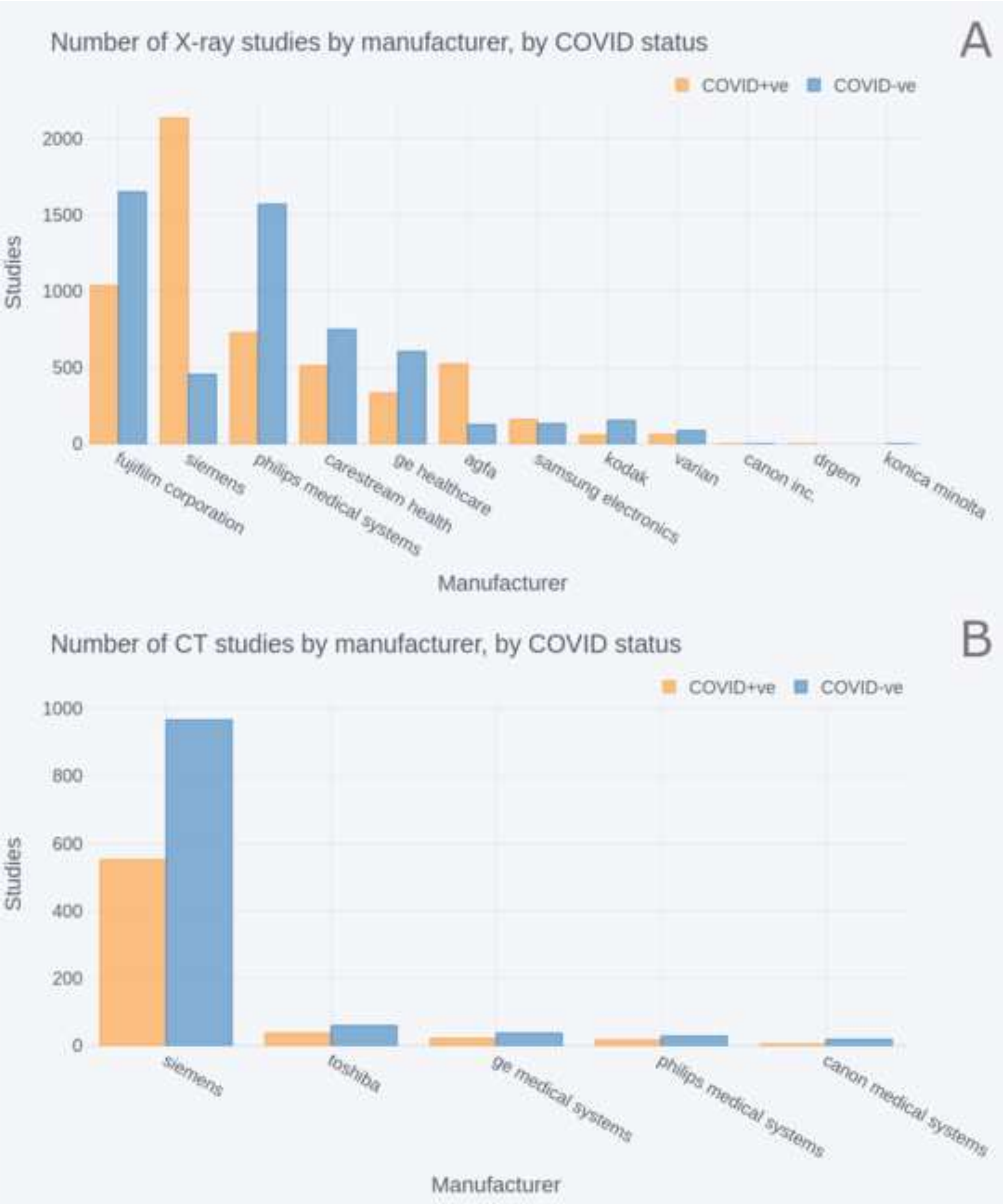

Number of X-ray studies by manufacturer, by COVID status
A
COVID+ve
COVID-ve
Studies
2000
1500
1000
500
0
fujifilm corporation
siemens
philips medical systems
carestream health
ge healthcare
agfa
samsung electronics
kodak
varian
canon inc.
drgem
konica minolta
Manufacturer
Number of CT studies by manufacturer, by COVID status
B
COVID+ve
COVID-ve
Studies
1000
800
600
400
200
0
siemens
toshiba
ge medical systems
philips medical systems
canon medical systems
Manufacturer

 Click here to access/download;Figure;fig6-maps.pdf

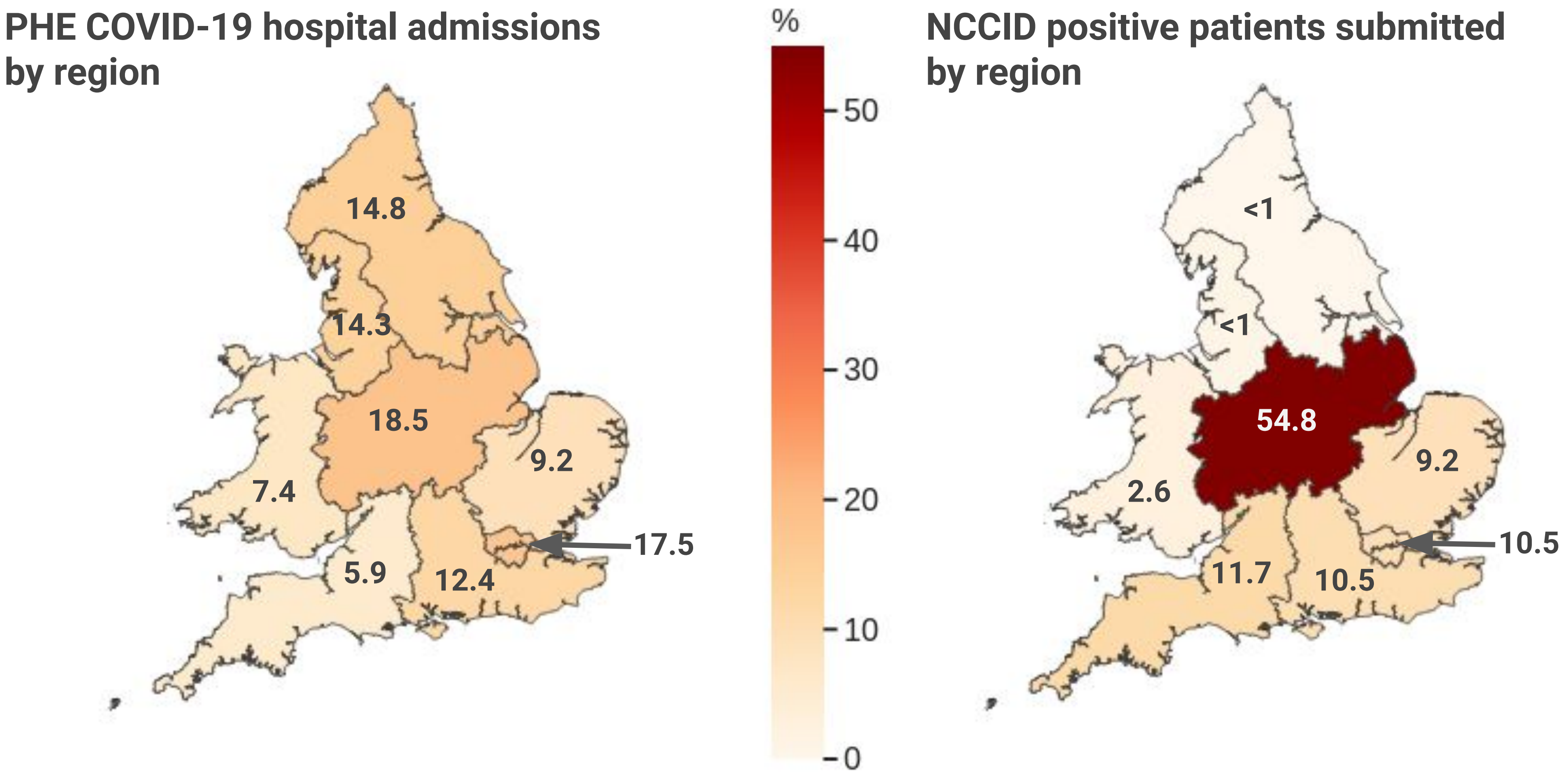

 Click here to access/download;Figure;fig6-regional.png

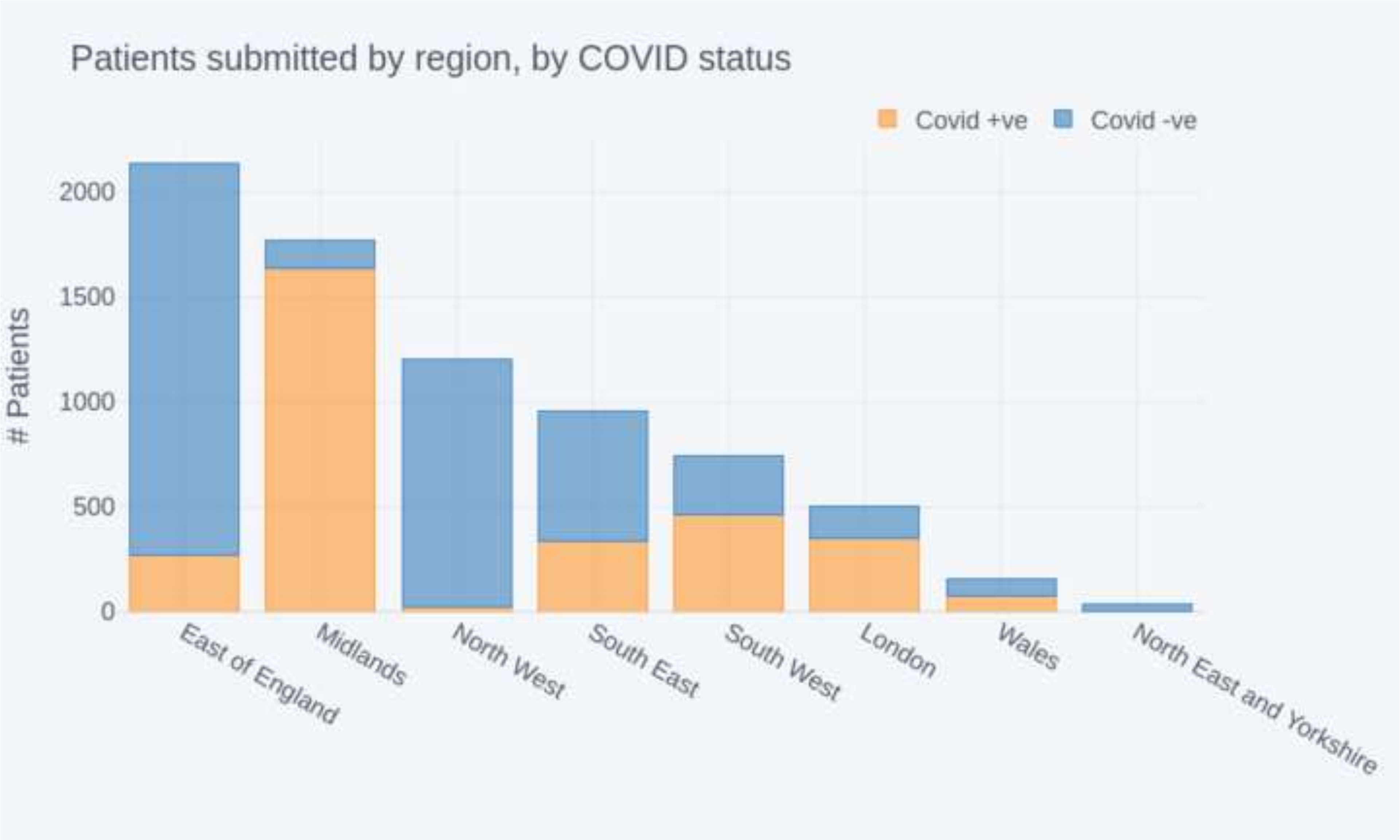

manuscript figure

Click here to access/download;Figure;fig7-sex.png

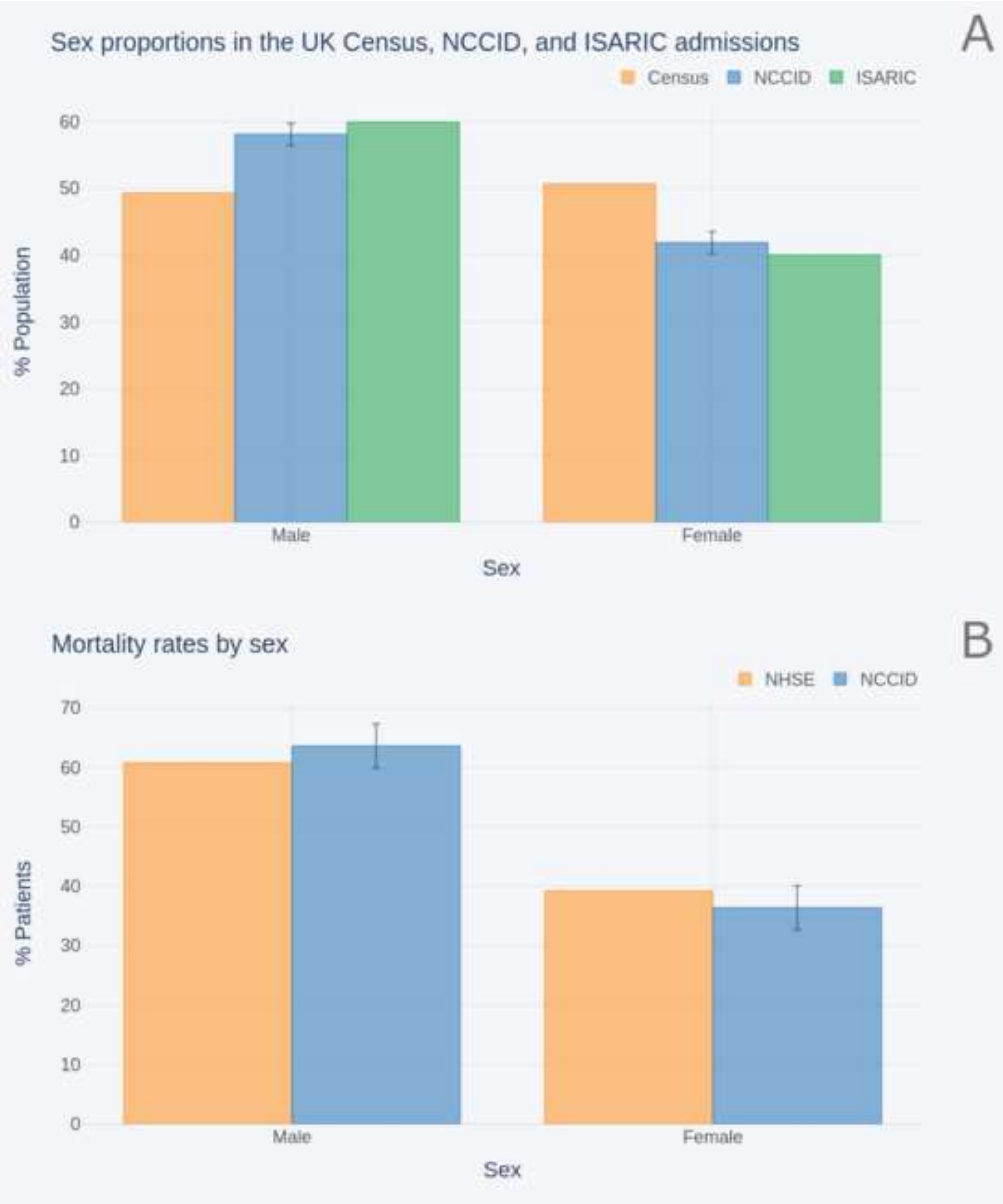

manuscript figure
Click here to access/download;Figure;fig8-ethnicity.png

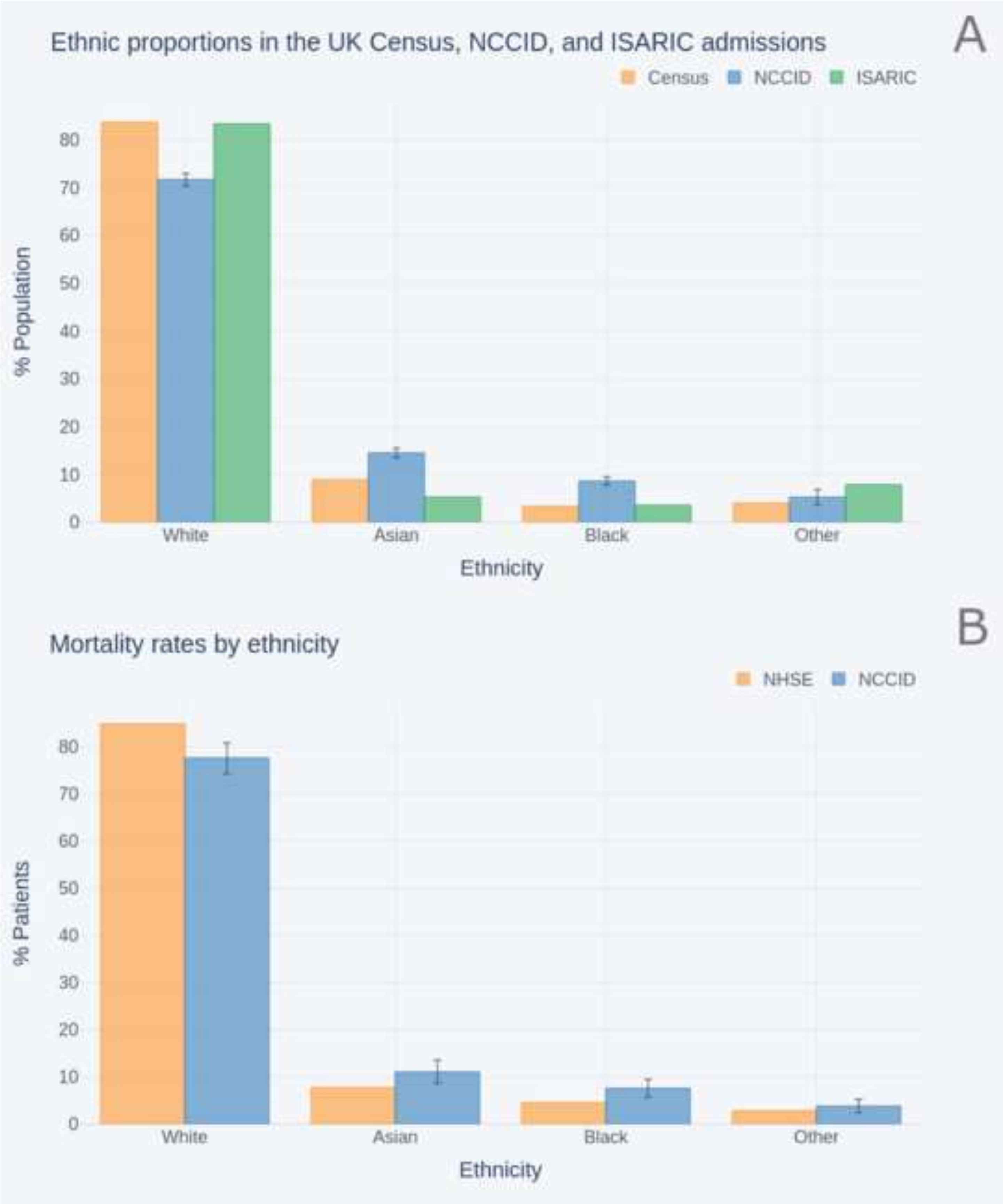

Manuscript Click here to access/download;Figure;fig9-age1.png

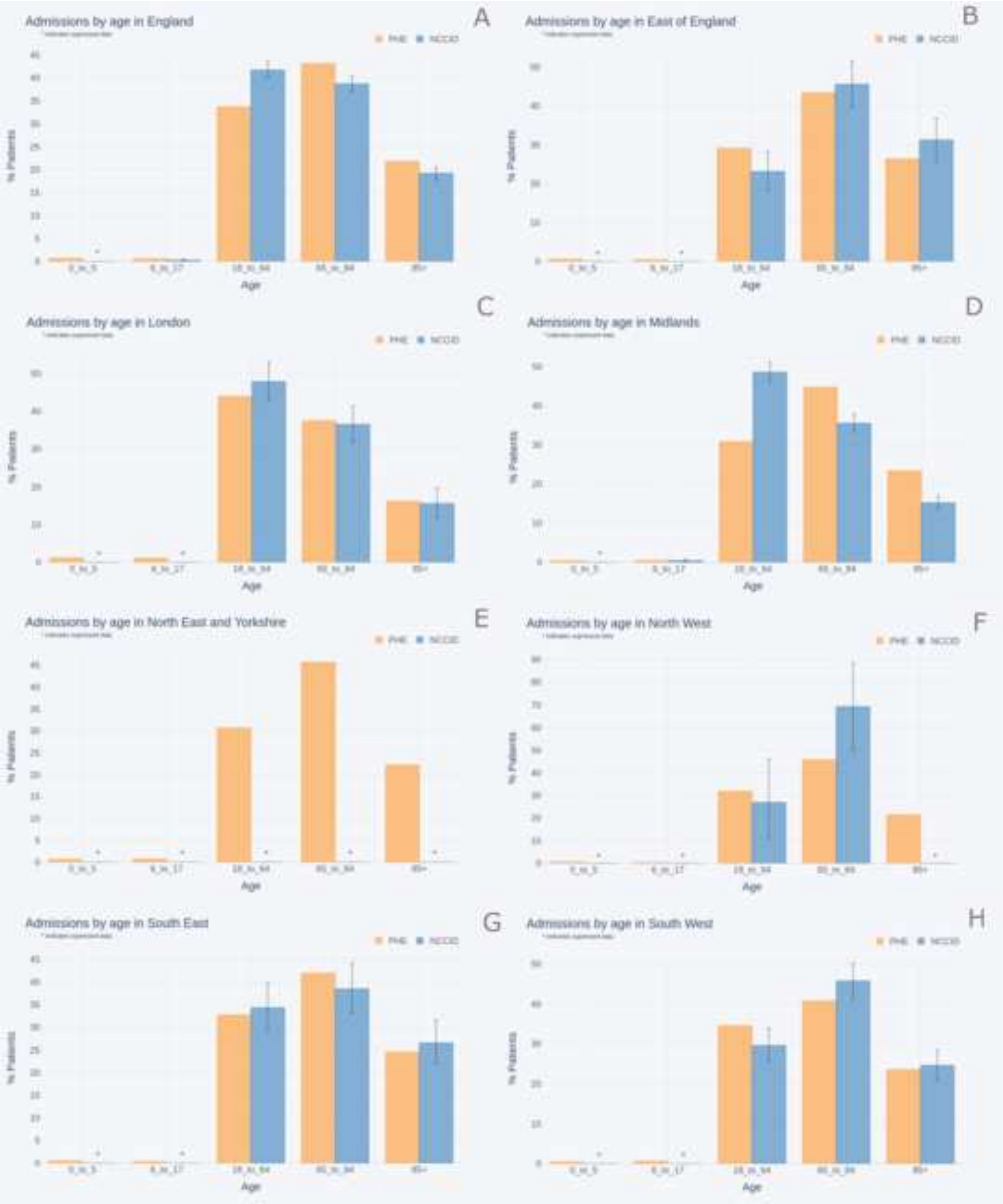

manuscript figure Click here to access/download;Figure;fig10-age2.png

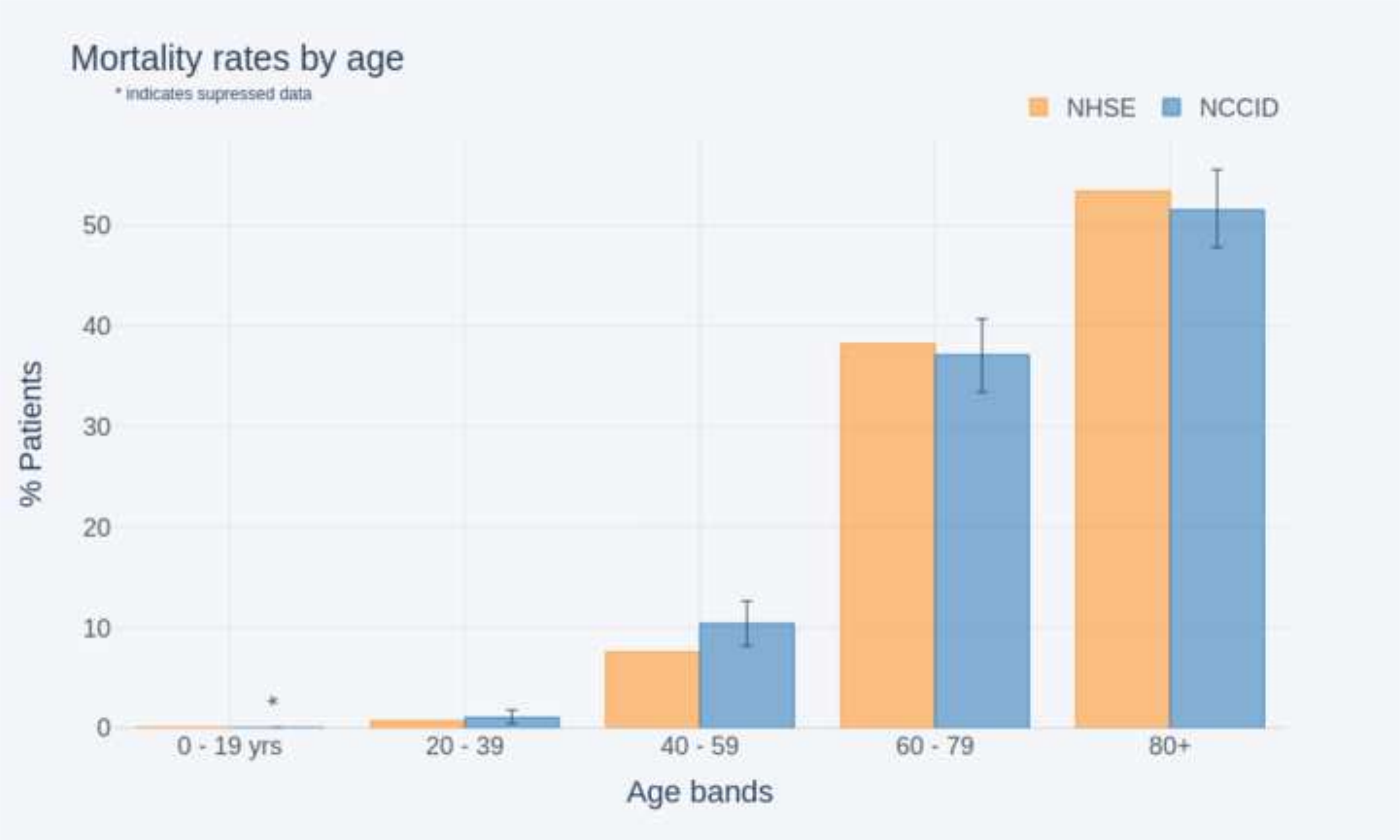

 Click here to access/download;Figure;fig11-temporal.png

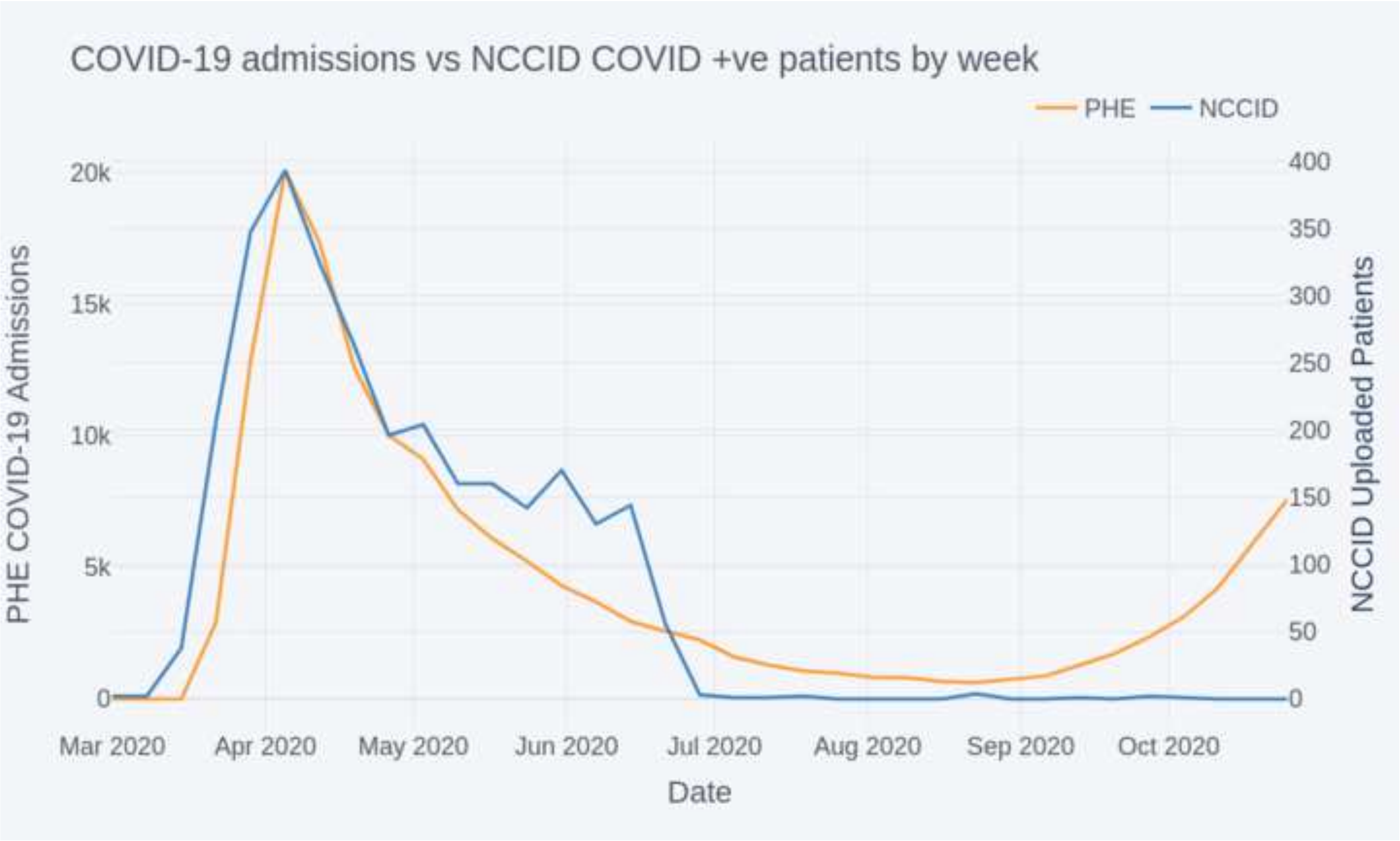

appendix

Click here to access/download
Supplementary Material
Appendix.pdf
