# Supplementary material for: An overview of the National COVID-19 Chest Imaging Database: data quality and cohort analysis
Source: Gigascience. 2021 Nov 25;10(11):giab076. doi: 10.1093/gigascience/giab076 (PMC8633457; doi:10.1093/gigascience/giab076)
Supplement: giab076_Online_Appendix [file giab076_online_appendix.pdf]

Appendix

A. Glossary

Imaging study – a study is either an X-ray, CT or MRI examination. Studies may contain multiple images (series) if i.e., several projections, body positions, fields of view, contrast types etc., were acquired during the appointment, but all of these individual images (image volumes for 3D modalities) are all amalgamated to a single data point in time – a study. Each study is assigned a unique accession number on PACS.

Site Definition – when referring to a 'site' in this paper we are talking about a Submitting Centre for NCCID. This is a Trust in England, or a Health Board in Wales. A single Trust or a Health Board can contain many different contributing hospital sites.

Sites contributing as of 29th October 2020:

- Ashford and St Peter's Hospitals NHS Foundation Trust
- Betsi Cadwaladr University Health Board
- Brighton and Sussex University Hospitals NHS Trust
- Cambridge University Hospitals NHS Foundation Trust
- Cwm Taf Morgannwg University Health Board
- George Eliot Hospital NHS Trust
- Hampshire Hospitals NHS Foundation Trust
- Imperial College Healthcare NHS Trust
- Liverpool Heart and Chest Hospital NHS Foundation Trust
- London North West University Healthcare NHS Trust
- NorfolkNorfolk and Norwich University Hospitals NHS Foundation Trust
- Oxford University Hospitals NHS Foundation Trust
- Royal Cornwall Hospitals NHS Trust
- Royal Surrey NHS Foundation Trust
- Royal United Hospitals Bath NHS Foundation Trust
- Sandwell and West Birmingham NHS Trust
- Sheffield Children's NHS Foundation Trust
- Somerset NHS Foundation Trust
- West Suffolk NHS Foundation Trust

The NCCID are continually onboarding new sites, a up to date list of submitters is available on the NCCID information page.

B. Scanner and image types

Manufacturer Models
The full list of model types for X-ray and CT across all imaging available in the dataset are included in Tables B.1 and B.2 in the Appendix where counts below 100, have been suppressed for brevity but all the model names have been listed below.

A single model type accounts for all X-ray studies from the largest supplier: Siemens – fluorospot compact FD. For the second largest X-ray supplier, Fujifilm, the model attribute was universally missing, making it hard to determine if single or multiple models are present. Most other X-ray manufacturers had multiple models available in the dataset. In the case of CT studies, the dominant manufacturer (Siemens) was split across ten different models, two of which had more than 500 studies each.

X-ray counts below 100 from:
agfa – cr15-x, dx-g, dxd30_wireless, dxd40_1000c, pixium_4343e_csi
canon inc. – axiom-multix m, cxdi, cxdi control software ne
carestream health – classic cr, cr0850a, drx-1, elite cr
drgem – gxr system
ge healthcare – discovery xr656, geode platform, global 1 platform, wdr1
kodak – classic cr, cr850, cr950, cr975/cr0975, drx-evolution, elite cr
konica minolta – 0862, cs-7
philips medical systems – pcr eleva
samsung electronics – dgr-c26u2b/us, dgr-ma1ba1/wr, dgr2aaa1cae21w, gc85a, gm85basic, sdr-agc80/com
siemens – siemens fd-x
toshiba_mec – tfd-3000a1

CT counts below 100:
canon medical systems – aquilion one, aquilion prime sp
ge medical systems – discovery 710, discovery ct750 hd, lightspeed16, lightspeed vct, optima ct660, revolution ct, revolution evo
philips medical systems – brilliance 64, ict 256, ingenuity core 128, ingenuity ct
siemens – emotion 16 (2007), sensation 64, somatom edge plus, somatom go.top, symbia intevo 16
toshiba – aquilion, aquilion one, aquilion prime, aquilion prime sp

Table 1. Model Manufacturer Name (X-ray). Machines that are suspected to be portable have been highlighted in red.

Manufacturer - Model (X-ray)	Studies
siemens - fluorospot compact fd	5093
fujifilm corporation - missing	3560
philips medical systems - digitaldiagnost	2414
carestream health - drx-revolution	1050
agfa - 3543eze	719
philips medical systems - mobilediagnost wdr	538
ge healthcare - optima xr220	526
ge healthcare - discovery xr656 hd	277
philips medical systems - digitaldiagnost c90	249
kodak - cr850a	224
samsung electronics - gm85	211
carestream health - cr975/cr0975	188
carestream health - drx-evolution	179
samsung electronics - gr40cw	174
ge healthcare - definium 6000	168
varian - varian_4336	151
agfa - cr 35	143
kodak - dr 7500	132
agfa - cr30-x	117
agfa - cr 85	115
ge healthcare - thunder platform	110
varian - varian_4343r	110

Table 2. Model Manufacturer Name (CT)

Manufacturer - Model (CT)	Studies
siemens - somatom definition as	624
siemens - somatom definition flash	568
siemens - somatom definition as+	412
siemens - somatom drive	200
siemens - somatom definition edge	141
siemens - somatom perspective	109
siemens - somatom force	103

X-ray format

Table 3. The number of X-ray studies in Digital Radiography (DX) and Computed Radiography (CR) formats as identified from the Modality DICOM attribute (0008, 0060) for COVID positive and negative patients.

Modality (X-ray Studies)	Positive	Negative
DX (Digital Radiography)	5026	4056
CR (Computed Radiography)	6699	1476

Photometric Interpretation

Table 4. The number of MONOCHROME1 and MONOCHROME2 X-ray studies as identified by the Photometric Interpretation attribute (0028, 0004) for COVID positive and negative patients. Images identified as MONOCHROME1 will need to be inverted to be aligned with those with MONOCHROME2.

Photometric Interpretation (X-ray Studies)	Positive	Negative
MONOCHROME2	8254	2562
MONOCHROME1	3478	2971

View Position

Table 5. The number of X-ray studies by View Position attribute (0018, 5101) for COVID positive and negative patients e.g., Anterior-Posterior (AP), Posterior-Anterior (PA), Lateral (LL).

View Position (X-ray Studies)	Positive	Negative
Missing	6662	2692
AP	3076	1598
PA	2003	1394
OTHER	10	7

C. Clinical data summary statistics

	count	mean	std	min	25%	50%	75%	max
**apache_score_on_itu_arrival**	97	16.4361	8.18156	0	12	16	20	43
**creatinine_on_admission**	1530	119.463	121.541	0.067	68	86	119.75	1569
**crp_on_admission**	1486	102	98.9551	0	27.925	76	145	1310
**d-dimer_on_admission**	333	1268.26	4439.43	0.02	0.95	2.633	605	49531
**diastolic_bp**	2669	74.2296	13.7393	33	66	74	82	180
**duration_of_symptoms**	1058	6.362	8.6413	0	1	5	9	186
**ferritin**	609	74318.1	461939	1.1	250	698	1846	6.961e+06
**fiO2_percentage**	547	36.3144	28.5774	0	21	28	40	100
**fibrinogen__if_d-dimer_not_performed**	368	6.12103	3.89517	1	4.6	5.9	7.3825	72
**heart_rate_on_admission**	1313	91.0274	20.2362	33	77	89	103	189
**lymphocyte_count_on_admission**	1530	2.27093	25.8469	0.09	0.6	0.9	1.3	700
**news2_score_on_arrival**	1196	4.06657	3.12194	0	2	4	6	17
**o2_saturation**	*	*	*	*	*	*	*	*
**pao2**	1083	79.2103	32.8453	0	89	95	97	100
**platelet_count_on_admission**	1531	235.133	123.027	1.2	160	214	289	2400
**respiratory_rate_on_admission**	1312	22.5739	6.60553	0	18	20	25	63
**systolic_bp**	2670	128.483	22.8418	46	113	126	142	240
**temperature_on_admission**	1305	37.3076	1.11667	27.9	36.5	37.2	38	40.7
**troponin_i**	541	891.01	8286.65	2	9	20	80	172696
**troponin_t**	108	312.877	2406.31	2	11	22.5	55.25	25000
**urea_on_admission**	1520	8.95618	7.62231	1.1	4.7	6.75	10.5	99
**wcc_on_admission**	1533	9.31315	24.5685	0.3	5.1	7.4	10.5	938

Figure 1. Summary statistics for the admission metrics taken by the NCCID. Low number suppression is indicated with a * for fields that have less than 7 counts.
