# Supplementary material for: An overview of the National COVID-19 Chest Imaging Database: data quality and cohort analysis
Source: Gigascience. 2021 Nov 25;10(11):giab076. doi: 10.1093/gigascience/giab076 (PMC8633457; doi:10.1093/gigascience/giab076)
Supplement: giab076_Response_to_Reviewer_Comments_Original_Submission [file giab076_response_to_reviewer_comments_original_submission.pdf]

Author's Response To Reviewer Comments

Close

GigaScience Referee Feedback for “An overview of the National COVID-19 Chest Imaging Database: data quality and cohort analysis” by Cushnan et al.

Reviewer #1:

Comments
1) Abstract is not much convincing and informative. Please refine.
The abstract has been updated and refined to provide additional information.

2) What is the motivation of this work? Please include in the manuscript.
We have expanded the introduction to make the motivations for the NCCID clearer (see response to reviewer 2). However as the manuscript is first and foremost a data note, the focus is largely descriptive with the overarching aim of informing technical users of this resource how they can best utilise it, as stated in the abstract, introduction, and conclusion.

3) Author can provide more appealing block diagram for figure 1.

Unfortunately, as the reviewer is not specific about what they disliked about the diagram, we have made updates we feel improve the aesthetic - we have made several adjustments, such as enlarging the font, renaming collection sites to hospital sites, and other minor placement adjustments, to improved the readability of the diagram.

4) Inclusion Criteria section is bit ambiguous. How these certain criteria are decided? Justify.
Given the data is collected in a real world setting the criteria had to be somewhat loosely defined to accommodate practical constraints. We have included the following clarifications in the manuscript:
Included population is relevant for models used on suspected COVID-19 cases. Therefore as a proxy for “suspected of COVID-19” we took, “undergone RT PCR test”.
3-4 weeks after swab is in order to exclude people who have had imaging a substantial amount of time after their COVID-19 infection to only capture the imaging that is contextual to COVID-19.

5) How your manuscript is different from other manuscripts? Kindly include in manuscript.
We are a little unclear as to what the Reviewer is asking. Our manuscript is the only Data Note describing the NCCID in detail. There are no other manuscripts of this nature for this particular database. Compared to Jacobs et al. 2020, which introduces and motivates the NCCID project more broadly, this manuscript is targeted to technical users who wish to access the database for purposes of developing and validating software. This has been clarified in the Introduction section of the paper.
There are several data repositories of COVID-19 imaging, but the NCCID has several unique qualities. It is the only COVID-19 imaging database specific to the UK population. It is to our knowledge the only COVID-19 database with a hold-out validation dataset which can be used to validate computer algorithms developed to assess COVID-19 (diagnosis or disease severity or prognosis). The manuscript also describes in comprehensive detail (as highlighted by the second Reviewer in their very positive reviews) the components of the database so as to aid researchers who might wish to use the database.

6) Refine the discussion part.
We have reviewed and refined the manuscript.

7) There are few linguistic and grammatical errors. Please correct.
We have further reviewed the manuscript to address these errors.

8) Similarity index must be less than 10 percent
We believe this is the case for our manuscript.

Reviewer #2:

This excellent Data Note provides an overview of the National COVID-19 Chest Imaging Database (NCCID), which is a centralised repository that hosts DICOM format radiological imaging data relating to COVID-19. By the very nature of this resource these data have immense reuse potential. The NCCID is the first national initiative of its kind - led by NHSX, British Society of Thoracic Imaging, and the Royal Surrey NHS Trust and Faculty - and the database hosts approximately 20,000 thoracic imaging studies related to SARS-CoV2 admissions from 20 NHS Hospitals / Trusts across England and Wales. Of note, the NCCID is additionally registered on the Health Data Research UK platform, with a platinum metadata rating which is a commendable achievement.

As part of this review, I used the NCCID Data Access Agreement, NCCID Data Access Framework Contract, and NCCID Application Form to gain access to the NCCID Project WorkSpace. This WorkSpace utilises the very powerful and highly intuitive faculty.ai platform to run Jupyter Notebooks on a remote server where the NCCID data can be accessed. I was impressed that the faculty.ai platform allows very many different views of the NCCID data, for example one option was to view the data by Scanner Type. This is an important consideration from a deep learning reuse perspective as it is known that different X-ray / CT scanners can introduce different artefacts, and this can confound multisite analysis (for example see Badgeley et al., 2019, https://doi.org/10.1038/s41746-019-0105-1). I find that by NCCID organising the imaging data in this way particularly helpful for addressing this issue.

I was additionally impressed that the NHS Analytics Unit was willing to provide an Onboarding Session to help a naïve user navigate the faculty.ai platform more effectively, and to provide one-on-one tuition on how the interface can be used for image analysis. I used this session to explore the functionality of the DICOM viewer that can be used to preview NCCID thoracic images. A Javascript viewer enables a user to open DICOM images and explore the image histogram of intensity values and I see this as a useful means of assessing, for example, contrast stretching in radiological image data that has been submitted to NCCID. As a follow-up to this Onboarding Session, there is now the additional option to launch a static viewer that offers a higher quality preview image of NCCID DICOM data. I find this functionality exceptionally helpful as it enables an end-user to preview image data and to visually inspect, for example, glassy nodules in COVID-19 thoracic image data prior to data download. I thank the NHS Analytics Unit for further developing the image visualisation capabilities of the NCCID Project WorkSpace as part of this review process. On this note I wish to highlight that, of the two viewers, I found the static viewer particularly helpful for assessing image quality of CT scans which was excellent.

I was further impressed that the thoracic imaging data includes a positive cohort with COVID-19, but also a negative cohort consisting of individuals with a negative swab test, but who may have a different underlying respiratory condition. This is an important consideration and it enables this dataset to be used for machine learning and deep learning approaches that could be used to distinguish between COVID-19 and other respiratory conditions in what remains a clinically relevant challenge.

Importantly, the code for the NCCID data warehouse and the Data Cleaning pipeline utilised in the paper are Open Source and available on GitHub (https://github.com/nhsx/covid-chest-imaging-database ; https://github.com/nhsx/nccid-cleaning) where they have been ascribed OSI-approved MIT licenses.

This is an excellent Data Note and I recommend this manuscript for publication in GigaScience.

We thank the reviewer for their positive appraisal of the manuscript and are pleased to hear that they enjoyed their experience whilst reviewing the database.

Minor comments

1. The MTA is tailored towards breast cancer screening. For example, there are the following definitions:
"Source Database" means the assembled collection of images collated from the research project entitled 'OPTIMAM: Optimisation of breast cancer detection using digital X-ray technology'.
"Related Data" means any and all pathological and clinical data associated with the Database Images supplied by or on behalf of CRT or Surrey to Company under this Agreement, in particular but without limitation, this may be identified regions of interest in the Database Images, the age of the woman at the date the relevant Database Image was taken, details about previous screening events, patient history, X-ray, ultrasound assessment, details of biopsy procedures and surgical events - all in a structured format representative in structure, format, quality, content and diversity of the Source

Database.

Can the authors please confirm that this MTA is suitable for thoracic radiology in the mixed sex COVID-19 study outlined in the accompanying preprint?

We would like to clarify that the example MTA was an outdated proposal for providing access to GigaScience and the reviewers. It was superseded by the data access request form and trusted research environment that Reviewer 2 went through instead. As such this document is no longer relevant to the NCCID and will not need adapting for future use.

2. In support of the manuscript, I further recommend that a copy of the NCCID Data Access Agreement, Data Access Framework Contract, Application Form, and snapshots of the code (GitHub archives) be archived in the GigaScience DataBase (GigaDB).

We are happy to provide these additional documents as supplementary resources alongside the manuscript. Regarding the codebase, as the data cleaning pipeline has versioned releases we have included the version numbers to the manuscript to ensure reproducibility.

Additional comments from another reviewer (unfinished review):

In general, it is a very detailed and comprehensive manuscript. However, I believe that the authors have exaggerated and overestimated the role of such databases in COVID-19 research, machine learning, diagnosis of COVID-19, and response to COVID-19 pandemic. At this stage, imaging is not used for "diagnosis" of COVID-19 and lab tests are available everywhere. Also, there are many imaging datasets similar to this one and I don't see anything special that distinguishes this one from others.

We agree with the reviewer that the role of imaging has changed throughout the course of the pandemic, and it is no longer used as a screening tool, due to the wider availability of lateral flow and PCR tests for diagnosis. However imaging is still clinically important in our understanding of COVID-associated pneumonia, its associated risk factors, as well as assessments of severity/prognosis. It may also prove useful in understanding symptomatic differences between variants of the COVID-19 virus as the NCCID continues to collect data.

A more long term goal of the authors is to provide a high-quality clinical database that the machine learning community can leverage as a research tool. Whilst the Reviewer is correct that the application of ML for diagnosis of COVID-19 infection is no longer medically useful, the pandemic does provide an interesting test bed for developing such technologies which we envisage will result in many useful learnings such as those already highlighted in Roberts et al, 2021. (https://doi.org/10.1038/s42256-021-00307-0)

We have included these wider motivations in the paper and clarified that diagnosis refers to the diagnosis of covid-associated acute respiratory syndrome rather than the diagnosis of a COVID-19 infection.

In the abstract the authors claim that "The National COVID-19 Chest Imaging Database (NCCID) is a centralised database containing chest X-rays, Computed Tomography (CT) scans and cardiac Magnetic Resonance Images (MRI) from patients across the UK" but I believe this contradict their later statement by saying "Only a small number of MRIs, 17, have been submitted, therefore MRI data is excluded from further analysis"

As the reviewer correctly points out, MRIs form only a small fraction of the data available in the NCCID and are certainly not available in large enough volumes to build models. We have therefore removed the above mention of them from the abstract to avoid the contradiction, though the existence of the small number of MRIs is still mentioned in the database overview section for completeness.

In the "clinical data" section, I have a concern regarding "ii. Important dates - such as swab dates, image dates and date of admission." I understand that the authors are from UK and I'm not familiar with their patient information privacy policies. However, in the US, according to HIPPA, these dates could be

patient identifiers. “All elements of dates (except year) for dates that are directly related to an individual, including birth date, admission date, discharge date, death date, and all ages over 89 and all elements of dates (including year) indicative of such age, except that such ages and elements may be aggregated into a single category of age 90 or older” so these could be patient identifiers and in the US would be taken into account in de-identification of the data.

This is a very important point and it is likely there are differences between the UK and US regarding the above mentioned information. We would like to reassure the reviewer that all fields being collected have been reviewed by IG experts within the NHS and have received ethical approval by the UK Research Authority. We have added this statement in the manuscript to avoid concerning future readers.

Moreover, the appropriate anonymisation level of the dataset is currently under review as the existing notice to collect data during the COVID pandemic is expiring in the UK in September 2021. As a result, additional abstraction methods may be implemented in the future while limiting as much as possible the impact on utility and the data users’ existing data processing pipelines.

Some minor comments:

COVID-19 stands for Coronavirus disease 2019 so COVID-19 disease is not a correct phrase to use.

This error has been rectified in the manuscript.

“v. COVID information, pertaining to how the patient was treated (intubation, admitted to ITU)”
All of the acronyms should be spelled out at the first mention like ITU in this sentence.

This has been rectified for the above example and other instances found in the manuscript.

In the "Medical history" section, “The presence of cardiovascular disease (CVS) and chronic kidney diseases (CKD) were both reported for approximately 90% of patients” This sentence is misleading. The first time I read it, I thought 90% of the patients had CVS and CKD which Is impossible. I believe the authors meant the presence or absence of … were reported.

This error has been rectified in the manuscript.

On top of these comments, please register any new software application in the bio.tools and SciCrunch.org databases to receive RRID (Research Resource Identification Initiative ID) and biotoolsID identifiers, and include these in your manuscript. This will facilitate tracking, reproducibility and re-use of your tool.
We agree that registering our tools on these platforms is a great idea, however it may take several weeks to acquire the necessary permissions due to NHS procedures. We are happy to pursue this but request for this to not be a requirement of publication. In the meantime, we have registered the project with a similar initiative to the ones mentioned above (the Health Data Research UK).

Close
