# Supplementary material for: An overview of the National COVID-19 Chest Imaging Database: data quality and cohort analysis
Source: Gigascience. 2021 Nov 25;10(11):giab076. doi: 10.1093/gigascience/giab076 (PMC8633457; doi:10.1093/gigascience/giab076)
Supplement: giab076_Response_to_Reviewer_Comments_Revision_1 [file giab076_response_to_reviewer_comments_revision_1.pdf]

Author's Response To Reviewer Comments

Close

comments have been addressed on the attached revision

Close
