# Supplementary material for: An overview of the National COVID-19 Chest Imaging Database: data quality and cohort analysis
Source: Gigascience. 2021 Nov 25;10(11):giab076. doi: 10.1093/gigascience/giab076 (PMC8633457; doi:10.1093/gigascience/giab076)
Supplement: giab076_Reviewer_2_Report_Original_Submission — Chris Armit -- 5/31/2021 Reviewed [file giab076_reviewer_2_report_original_submission.pdf]

Reviewer Report

Title: An overview of the National COVID-19 Chest Imaging Database: data quality and cohort analysis

Version: Original Submission **Date:** 5/31/2021

Reviewer name: Chris Armit

Reviewer Comments to Author:

This excellent Data Note provides an overview of the National COVID-19 Chest Imaging Database (NCCID), which is a centralised repository that hosts DICOM format radiological imaging data relating to COVID-19. By the very nature of this resource these data have immense reuse potential. The NCCID is the first national initiative of its kind - led by NHSX, British Society of Thoracic Imaging, and the Royal Surrey NHS Trust and Faculty - and the database hosts approximately 20,000 thoracic imaging studies related to SARS-CoV2 admissions from 20 NHS Hospitals / Trusts across England and Wales. Of note, the NCCID is additionally registered on the Health Data Research UK platform, with a platinum metadata rating which is a commendable achievement. As part of this review, I used the NCCID Data Access Agreement, NCCID Data Access Framework Contract, and NCCID Application Form to gain access to the NCCID Project WorkSpace. This WorkSpace utilises the very powerful and highly intuitive faculty.ai platform to run Jupyter Notebooks on a remote server where the NCCID data can be accessed. I was impressed that the faculty.ai platform allows very many different views of the NCCID data, for example one option was to view the data by Scanner Type. This is an important consideration from a deep learning reuse perspective as it is known that different X-ray / CT scanners can introduce different artefacts, and this can confound multisite analysis (for example see Badgeley et al., 2019, https://doi.org/10.1038/s41746-019-0105-1). I find that by NCCID organising the imaging data in this way particularly helpful for addressing this issue.I was additionally impressed that the NHS Analytics Unit was willing to provide an Onboarding Session to help a naÃ¯ve user navigate the faculty.ai platform more effectively, and to provide one-on-one tuition on how the interface can be used for image analysis. I used this session to explore the functionality of the DICOM viewer that can be used to preview NCCID thoracic images. A Javascript viewer enables a user to open DICOM images and explore the image histogram of intensity values and I see this as a useful means of assessing, for example, contrast stretching in radiological image data that has been submitted to NCCID. As a follow-up to this Onboarding Session, there is now the additional option to launch a static viewer that offers a higher quality preview image of NCCID DICOM data. I find this functionality exceptionally helpful as it enables an end-user to preview image data and to visually inspect, for example, glassy nodules in COVID-19 thoracic image data prior to data download. I thank the NHS Analytics Unit for further developing the image visualisation capabilities of the NCCID Project WorkSpace as part of this review process. On this note I wish to highlight that, of the two viewers, I found the static viewer particularly helpful for assessing image quality of CT scans which was excellent.I was further impressed that the thoracic imaging data includes a positive cohort with COVID-19, but also a negative cohort consisting of individuals with a negative swab test, but who may have a different underlying respiratory condition. This is an important consideration and it enables this dataset to be used for machine learning and deep

learning approaches that could be used to distinguish between COVID-19 and other respiratory conditions in what remains a clinically relevant challenge.Importantly, the code for the NCCID data warehouse and the Data Cleaning pipeline utilised in the paper are Open Source and available on GitHub (https://github.com/nhsx/covid-chest-imaging-database ; https://github.com/nhsx/nccid-cleaning) where they have been ascribed OSI-approved MIT licenses.This is an excellent Data Note and I recommend this manuscript for publication in GigaScience.Minor comments1. The MTA is tailored towards breast cancer screening. For example, there are the following definitions:"Source Database" means the assembled collection of images collated from the research project entitled 'OPTIMAM: Optimisation of breast cancer detection using digital X-ray technology'."Related Data" means any and all pathological and clinical data associated with the Database Images supplied by or on behalf of CRT or Surrey to Company under this Agreement, in particular but without limitation, this may be identified regions of interest in the Database Images, the age of the woman at the date the relevant Database Image was taken, details about previous screening events, patient history, X-ray, ultrasound assessment, details of biopsy procedures and surgical events - all in a structured format representative in structure, format, quality, content and diversity of the Source Database. Can the authors please confirm that this MTA is suitable for thoracic radiology in the mixed sex COVID-19 study outlined in the accompanying preprint?2. In support of the manuscript, I further recommend that a copy of the NCCID Data Access Agreement, Data Access Framework Contract, Application Form, and snapshots of the code (GitHub archives) be archived in the GigaScience DataBase (GigaDB).

Level of Interest

Please indicate how interesting you found the manuscript: Choose an item.

Quality of Written English

Please indicate the quality of language in the manuscript: Choose an item.

Declaration of Competing Interests

Please complete a declaration of competing interests, considering the following questions:

- Have you in the past five years received reimbursements, fees, funding, or salary from an organisation that may in any way gain or lose financially from the publication of this manuscript, either now or in the future?
- Do you hold any stocks or shares in an organisation that may in any way gain or lose financially from the publication of this manuscript, either now or in the future?
- Do you hold or are you currently applying for any patents relating to the content of the manuscript?
- Have you received reimbursements, fees, funding, or salary from an organization that holds or has applied for patents relating to the content of the manuscript?
- Do you have any other financial competing interests?

- Do you have any non-financial competing interests in relation to this paper?

If you can answer no to all of the above, write 'I declare that I have no competing interests' below. If your reply is yes to any, please give details below.

I work for GigaScience where I perform checks on the quality of image data. My funding is not dependent on the outcome of this review, and I declare that I have no competing interests.

I agree to the open peer review policy of the journal. I understand that my name will be included on my report to the authors and, if the manuscript is accepted for publication, my named report including any attachments I upload will be posted on the website along with the authors' responses. I agree for my report to be made available under an Open Access Creative Commons CC-BY license (http://creativecommons.org/licenses/by/4.0/). I understand that any comments which I do not wish to be included in my named report can be included as confidential comments to the editors, which will not be published.

Choose an item.

To further support our reviewers, we have joined with Publons, where you can gain additional credit to further highlight your hard work (see: https://publons.com/journal/530/gigascience). On publication of this paper, your review will be automatically added to Publons, you can then choose whether or not to claim your Publons credit. I understand this statement.

Yes Choose an item.
