# Supplementary material for: An overview of the National COVID-19 Chest Imaging Database: data quality and cohort analysis
Source: Gigascience. 2021 Nov 25;10(11):giab076. doi: 10.1093/gigascience/giab076 (PMC8633457; doi:10.1093/gigascience/giab076)
Supplement: giab076_Reviewer_2_Report_Revision_1 — Chris Armit -- 8/20/2021 Reviewed [file giab076_reviewer_2_report_revision_1.pdf]

Reviewer Report

Title: An overview of the National COVID-19 Chest Imaging Database: data quality and cohort analysis

Version: Revision 1 **Date:** 8/20/2021

Reviewer name: Chris Armit

Reviewer Comments to Author:

This excellent Data Note describes the National COVID-19 Chest Imaging Database (NCCID). This fantastic resource hosts approximately 20,000 thoracic imaging studies related to SARS-CoV2 admissions from 20 NHS Hospitals / Trusts across England and Wales, and additionally includes the NCCID Project WorkSpace which enables researchers to analyse these data on a remote server where the NCCID data can be accessed. The manuscript is well-written, and in their response the authors have addressed all my comments and have clarified that the MTA form has been replaced by the data access request form and trusted research environment that I used during the review process. In addition, the authors have addressed the comments from the other reviewers. I applaud the authors for highlighting the clinical significance of imaging for understanding COVID-associated pneumonitis. In addition, I thank the authors for further highlighting that this manuscript "is the only Data Note describing the NCCID in detail" and that "there are no other manuscripts of this nature for this particular database".The NCCID is an outstanding resource, and I anticipate it being used widely by the biomedical research community. This is an exceptional Data Note and I recommend this manuscript for publication in GigaScience.

Level of Interest

Please indicate how interesting you found the manuscript: Choose an item.

Quality of Written English

Please indicate the quality of language in the manuscript: Choose an item.

Declaration of Competing Interests

Please complete a declaration of competing interests, considering the following questions:

- Have you in the past five years received reimbursements, fees, funding, or salary from an organisation that may in any way gain or lose financially from the publication of this manuscript, either now or in the future?
- Do you hold any stocks or shares in an organisation that may in any way gain or lose financially from the publication of this manuscript, either now or in the future?
- Do you hold or are you currently applying for any patents relating to the content of the manuscript?

- Have you received reimbursements, fees, funding, or salary from an organization that holds or has applied for patents relating to the content of the manuscript?
- Do you have any other financial competing interests?
- Do you have any non-financial competing interests in relation to this paper?

If you can answer no to all of the above, write 'I declare that I have no competing interests' below. If your reply is yes to any, please give details below.

I work for GigaScience where I perform checks on the quality of image data and the image analysis approach. My funding is not dependent on the outcome of this review, and I declare that I have no competing interests.

I agree to the open peer review policy of the journal. I understand that my name will be included on my report to the authors and, if the manuscript is accepted for publication, my named report including any attachments I upload will be posted on the website along with the authors' responses. I agree for my report to be made available under an Open Access Creative Commons CC-BY license (http://creativecommons.org/licenses/by/4.0/). I understand that any comments which I do not wish to be included in my named report can be included as confidential comments to the editors, which will not be published.

Choose an item.

To further support our reviewers, we have joined with Publons, where you can gain additional credit to further highlight your hard work (see: https://publons.com/journal/530/gigascience). On publication of this paper, your review will be automatically added to Publons, you can then choose whether or not to claim your Publons credit. I understand this statement.

Yes Choose an item.
